# Ankle osteoarthritis and its association with severe ankle injuries, ankle surgeries and health-related quality of life in recently retired professional male football and rugby players: a cross-sectional observational study

**DOI:** 10.1136/bmjopen-2020-036775

**Published:** 2020-06-21

**Authors:** Liam D. A. Paget, Haruhito Aoki, Simon Kemp, Mike Lambert, Clint Readhead, Keith A Stokes, Wayne Viljoen, Gustaaf Reurink, Johannes L Tol, Gino M M J Kerkhoffs, Vincent Gouttebarge

**Affiliations:** 1 Orthopaedic Surgery, Amsterdam UMC - Locatie AMC, Amsterdam, North Holland, The Netherlands; 2 Academic Center for Evidence-based Sports Medicine (ACES), Amsterdam, The Netherlands; 3 Amsterdam Collaboration for Health and Safety in Sports, AMC/VUMC IOC Research Center (ACHSS), Amsterdam, The Netherlands; 4 St Marianna University School of Medicine, Kawasaki, Japan; 5 Yokohama City Sports Medical Center, Yokohama, Japan; 6 Rugby Football Union, Twickenham, UK; 7 Division of Exercise Science and Sports Medicine (ESSM), University of Cape Town, Cape Town, Western Cape, South Africa; 8 South African Rugby Union (SARU), Cape Town, Western Cape, South Africa; 9 Department for Health, University of Bath, Bath, United Kingdom; 10 Centre for Sport, Exercise and Osteoarthritis Research versus Arthritis, Nottingham, United Kingdom; 11 Sports Medicine, The Sport Physician Group, OLVG, Amsterdam, The Netherlands; 12 Aspetar Orthopaedic and Sports Medicine Hospital, Doha, Ad Dawhah, Qatar; 13 Football Players Worldwide (FIFPRO), Hoofddorp, The Netherlands

**Keywords:** epidemiology, orthopaedic & trauma surgery, foot & ankle, orthopaedic sports trauma, sports medicine

## Abstract

**Objectives:**

To determine (1) the prevalence of ankle osteoarthritis (OA) among former professional football and rugby players, (2) assess the association between ankle injuries or ankle surgeries with ankle OA, and (3) compare the mental and physical quality of life (QoL) between former professional football and rugby players with and without OA.

**Methods:**

We conducted a questionnaire-based observational study with a cross-sectional design. Former professional football and rugby players were recruited by the Football Players Worldwide and the International Rugby Players. Information concerning ankle OA, sustained ankle injuries and ankle surgeries was gathered (medical record or most recent medical professional). Health-related QoL was assessed using the Patient-Reported Outcomes Measurement Information System (PROMIS) physical and mental health scores.

**Results:**

Overall, 553 former professional football (n=401) and rugby (n=152) players were enrolled in the study (response rate of 56%). Ankle OA prevalence among former professional football and rugby players was 9.2% and 4.6%, respectively. Football players were more likely to suffer from ankle OA following every ankle injury and/or surgery. Football and rugby players with ankle OA had similar PROMIS physical and mental health scores to the norm for the general population.

**Conclusion:**

Former professional football and rugby players had higher ankle OA prevalence than the general population (3.4%). Football players are more likely to suffer from ankle OA following every ankle injury and/or surgery. No clinically relevant difference was seen for physical or mental health-related QoL among football and rugby players. Preventive measures for ankle injuries are recommended.

**Strengths and limitations of this study**

- The study had overall good response rate (56%) and a large group of participating football players (n=401).
- This study had a cross-sectional design; therefore, no causal association can be determined.
- This study contributes to the literature where the currently available data concerning ankle osteoarthritis in professional football and rugby players are outdated or non-existent.
- The data are based on (retrospective) questionnaires and are therefore dependent on the accuracy and understanding of the participant.
- Our definition of osteoarthritis did not include a clinical and radiological evaluation.

## Background

Football and rugby union (hereafter ‘rugby’) are among the most popular team sports worldwide. In both sports, ankle injuries represent a significant proportion of the total injuries sustained.^1–5^ Previous ankle injuries are an important risk factor for ankle osteoarthritis (OA).^6 7^

Drawer and Fuller^8^ estimate the overall risk of injury in professional football to be 1000 times higher than in high-risk industrial occupations, with ankle injuries contributing 10%–18% of all injuries.^2^ The Union of European Football Associations Champions League Injury Study reported an overall ankle injury rate of 1 injury per 1000 hours.^2^ Lateral ankle sprains (51%) were the most prevalent subtype of ankle injuries.^2^ In professional rugby, epidemiological studies show that ankle injuries contribute 5%–20% of all injuries.^1 3 9 10^ Similar to professional football, lateral ankle sprains are the most common ankle injuries, with an injury rate of 3.8–4.5 injuries per 1000 player hours depending on the position on the field.^11^

Two retrospective cohort studies from the general population (n=390 and n=639 patients, respectively) reported that 70%–78% of ankle OA cases were associated with a previously sustained ankle injury. These previous injuries included fractures, isolated osteochondral defects of the talus and ankle ligament injuries.^6 7^ Among these post-traumatic ankle OA cases, 28% were associated with a previously sustained ligamentous injury.^7^ To our knowledge, there are no prospective studies on the association of ankle injuries and ankle OA.

In the general population the prevalence of ankle OA is estimated to be 3.4% in people over 50 years of age.^12^ The reported prevalence of ankle OA 16–22 years after a professional football career is 12%–19%.^13–15^ No data are available for ankle OA among former professional rugby players. In comparison with a general population control group, the prevalence of OA in any joint and the incidence of joint replacement for any joint were estimated to be four and six times higher, respectively, among former elite rugby players.^16^ This has been associated with a significantly poorer mental and physical health-related quality of life (QoL).^16^

Several other studies report the association of general OA with mental health symptoms and physical health-related QoL among former elite athletes across professional team sports including football and rugby.^17 18^ Therefore, the impact of OA on retired players must be considered. To date, the epidemiological evidence concerning the prevalence of ankle OA in professional football is either outdated or limited, and in the case of rugby non-existent, while data on the association between previous ankle injury and ankle OA are scarce in both sports.

The objective of this study was threefold. First is to evaluate the prevalence of ankle OA in recently retired professional football and rugby players. Second is to determine the association between ankle injury and/or ankle surgery and ankle OA. Third is to compare the mental and physical QoL in players with and without ankle OA. Our hypotheses were that (1) recently retired professional football and rugby players have higher ankle OA prevalence than the general population (as found in the literature); (2) ankle injuries and/or ankle surgeries are associated with ankle OA; and (3) the mental and physical QoL of recently retired professional football and rugby players with OA are lower compared with players without OA.

## Methods

### Design

We conducted a questionnaire-based observational study with a cross-sectional design. The study was reported according to the ‘Strengthening the Reporting of Observational Studies in Epidemiology’ statement.^19^

### Participants

Retired professional football and rugby players were randomly recruited to reduce recruitment bias by the Football Players Worldwide (FIFPRO) and the International Rugby Players (IRP). Inclusion criteria were (1) being a retired male professional football or rugby player; (2) being younger than 50 years of age; and (3) being able to read and comprehend texts in English, French or Spanish.

The definition of a retired professional football or rugby player was that he (1) has trained to improve performances, (2) has competed in the highest or second highest national league, and (3) has had training and competition as a major activity (way of living) or focus of personal interest, devoting several hours in all or most of the days for these activities, and exceeding the time allocated to other types of professional or leisure activities. If interested, players received information about the study, gave their informed consent and completed an electronic questionnaire. Once completed, the electronic questionnaires were saved automatically on a secured electronic server that only the principal investigator could access.

Sample size calculation for ankle OA prevalence indicated that 138 participants per sport were needed (power of 80%, CI of 95%, absolute precision of 5%) under the assumption of an anticipated population proportion of 10%.^20^ Expecting a response rate of approximately 50%, we intended to reach at least 300 participants per sport.

### Dependent variable: ankle OA

The clinical diagnosis of ankle OA by a medical professional was retrospectively determined using a single question (‘Have you been diagnosed with ankle osteoarthritis by a medical professional?’). Participants were given the definition of ankle OA (based on the National Institute for Health and Care Excellence criteria; adapted for age), determined as the damage of the ankle joint’s cartilage that leads to activity-related joint pain with either no morning joint-related stiffness or morning stiffness that lasts no longer than 30 min.^21^ For this question, participants were requested to consult either their medical record or their most recent medical professional.

### Independent variable: severe ankle injury and related surgery

History of a severe ankle injury during their professional football or rugby career was examined through a single question for the sequential recording of single or multiple injury events over time. Similarly, history of an ankle-related surgery was recorded through a single question for the sequential recording of a single or multiple ankle surgeries over time. In our study, a severe ankle injury was defined as an injury that involved the ankle joint, occurred during team activities (training or match), and led to absence from either training or a match for more than 28 days.^18 19^ For this question, participants were requested to consult either their medical records or their most recent medical professional.

### Health-related QoL

The Patient-Reported Outcomes Measurement Information System Global Health short form (PROMIS-GH) assesses multiple domains related to health-related QoL, such as health, functioning, pain, social activities and fatigue.^20^ The PROMIS-GH has been validated in several populations and languages, among which are English, French and Spanish (for detailed information, see www.nihpromis.org).^20^ The global mental health and global physical health scores were calculated based on 10 items each measured on a 5-point scale (from 1 to 5) and subsequently converted.^20^ These subscale scores ranged from 0 to 100, with a higher score indicating better QoL and a mean score of 50 indicating the norm for the general population.^22^

### Procedures

An anonymous questionnaire (electronic and/or paper) available in English, French and Spanish was compiled (LimeSurvey Professional). It included the following descriptive variables: age, body height, body weight, duration of professional football or rugby career, level of play, duration and nature of retirement, and current employment status (employed or not employed). Additionally, this questionnaire included questions for the outcomes previously elaborated on (ankle OA, ankle injury, ankle surgery, QoL). Information about the study was sent per email to potential participants by FIFPRO and IRP. Participants interested in the study gave their informed consent and completed the electronic questionnaire. Participants were asked to complete the questionnaire within 2 weeks, with reminders being sent after 2 and 4 weeks. For privacy reasons, the responses to the questionnaires were coded and anonymised. Once completed, the electronic questionnaires were automatically saved on a secured electronic server that only the principal investigator could access. Players participated voluntarily and did not receive any reward for their participation.

### Statistical analyses

Data analysis was performed using the statistical software IBM SPSS V.24.0 for Windows. Analyses were conducted separately for retired professional football and rugby players. Data were assessed (visually) for normality and presented as mean±SD, median (IQR) or frequency (proportion in %), as appropriate. Body mass index (BMI) was calculated using the provided weight and height of participants (kg/m^2^). Prevalence of ankle OA, overall and within the age categories of ≤40 years and >40 years, was calculated as the proportion of the number of participants with ankle OA relative to the total number of participants in each category.^20^ We chose to dichotomise age at 40 years, as our population included former professional athletes. This is opposed to Murray *et al*^12^ concerning the general population (dichotomised at 50 years of age) and in line with Song *et al*^23^ studying former professional American football players. We made the assumption that although no longer professionals, these former professional athletes would no longer be competing at a decent level at the age of 40. We therefore found 40 years to be a more relevant age to dichotomise than 50 years. A logistic regression analysis was performed to determine the association of the number of severe ankle injuries and/or ankle surgeries (continuous independent variable) with ankle OA (dichotomous dependent variable). Severe ankle injuries and/or ankle-related surgeries were also expressed using three predetermined categories (0, 1 and >1). This was adjusted for age and BMI, both having been identified as risk factors for OA.^24 25^ Descriptive analyses of health-related QoL (global physical health and global mental health) were conducted, while comparisons between groups (retired players with ankle OA vs retired players without ankle OA) were made using Mann-Whitney U test for independent samples.^20^

### Patient and public involvement

This research was done without patient involvement. Patients were not invited to comment on the study design and were not consulted to develop patient-relevant outcomes or interpret the results. Patients were not invited to contribute to the writing or editing of this document for readability or accuracy.

## Results

### Participants

From a total of 750 football and 326 rugby players contacted, respectively, 401 and 152 gave their written informed consent and completed the questionnaire (overall response rate of 56%). The characteristics of the two groups, including the predetermined subcategories for severe ankle injuries and ankle surgeries (0, 1 and >1), are illustrated in table 1.

**Table 1:** Descriptive characteristics of recently retired professional football players and rugby players

	Football			Rugby		
	Total (n=401)	Ankle OA (n=37)	No ankle OA (n=364)	Total (n=152)	Ankle OA (n=7)	No ankle OA (n=145)
Age in years*, median (IQR; min–max)/mean±SD	36 (32–40; 25–50)	39 (36–42; 27–49)*	36 (32–40; 25–50)*	40±6	41±7	40±6
Height in cm, mean±SD/median (IQR; min–max)	181±7	180±7	182±7	186 (180–191; 166–203)	191 (173–192; 172–192)	186 (180–191; 166–203)
Weight in kg, mean±SD/median (IQR; min–max)	82±9	82±13	82±9	100 (91–114; 59–170)	108 (89–123; 82–130)	100 (91–113; 59–170)
BMI in kg/m^2^, median (IQR; min–max)	24.8 (23.5–26.2; 20.1–32.7)	24.8 (22.9–26.6; 20.3–29)	24.7 (23.5–26.1; 20.1–32.7)	29 (27.1–31.4; 20.4–52.5)	32.2 (28.5–33.7; 27.4–35.3)*	28.9 (27.0–31.2; 20.4–52.5)*
Employed in % (n)	89 (350)	92(33)	89 (317)	91 (139)	86 (6)	92 (133)
Professional career duration in years, median (IQR; min–max)	13 (10–15; 2–19)	13 (12–16; 5–18)	13 (9–15; 2–19)	10 (7–15; 2–20)	10 (8–12.5; 8–13)	10 (7–15; 2–20)
Retirement duration in years, median (IQR; min–max)	4 (2–7; 1–17)	5 (3–9; 1–12)	4 (2–7; 1–17)	8 (3–12; 0–22)	12 (2–16; 1–19)	8 (4–11; 0–22)
Level of play, top league, % (n)	81 (314)	92 (34)	80 (280)	80 (106)	86 (6)	80 (100)
Forced retirement, % (n)	29 (113)	27 (10)	30 (103)	36 (55)	57 (4)	35 (51)
Severe ankle injuries, median (IQR; min–max)	1 (0–2; 0–10)	2 (1–3; 0–10)	1 (0–2; 0–10)	1 (0–2; 0–20)	1 (1–2; 0–3)	1 (0–2; 0–20)
Ankle surgeries, median (IQR; min–max)	0 (0–0; 0–5)	1 (0–2; 0–5)	0 (0–0; 0–5)	0 (0–0; 0–4)	1 (0–1; 0–2)	0 (0–0; 0–4)

*Statistically significant difference between ankle OA and no ankle OA.

BMI, body mass index; max, maximum; min, minimum; n, number; OA, osteoarthritis.

### Prevalence of ankle OA, severe injury and surgery

The prevalence of patient-reported ankle OA (including the predetermined categories of ≤40 years and >40 years) among recently retired professional football and rugby players is presented in table 2. The prevalence of ankle OA in former professional football players was 9.2% (n=37). Overall, 94% of players with ankle OA were documented to have sustained ≥1 ankle injury and 62% had undergone ≥1 ankle surgery. In the non-OA group (n=364), 54% had sustained ≥1 ankle injury and 14% had undergone ≥1 ankle surgery. Logistic regression analysis (table 3) showed that football players who had sustained a severe ankle injury were 1.3 (95% CI 1.1 to 1.6) times more likely to report ankle OA (p<0.001). Football players who had undergone ankle surgery were 2.1 (95% CI 1.4 to 3.1) times more likely to report ankle OA (p<0.001).

**Table 2:** Subcategories for prevalence of reported ankle OA, severe injuries and ankle surgeries in retired football and rugby players

	Football			Rugby		
	Total (n=401)*	≤40 years (n=271)	>40 years (n=83)	Total (n=152)	≤40 years (n=82)	>40 years (n=66)
Prevalence ankle OA, % (95% CI)	9.2 (6.4 to 12.1)	8.5 (5.2 to 11.8)	12.0 (5.0 to 19.1)	4.6 (1.2 to 8.0)	2.4 (0.9 to 5.8)	4.5 (0.5 to 9.6)
	0 ankle injuries	1 ankle injury	>1 ankle injury	0 ankle injuries	1 ankle injury	>1 ankle injury
Severe ankle injuries total, % (n)	42.5 (156)	25.3 (93)	32.2 (118)	40.5 (60)	27.7 (41)	31.8 (47)
With ankle OA, % (n)	5.7 (2)	28.6 (10)	65.7 (23)	14.3 (1)	42.9 (3)	42.9 (3)
Without ankle OA, % (n)	46.4 (154)	25.0 (83)	28.6 (95)	41.8 (59)	27.0 (38)	31.2 (44)
	No ankle surgeries	1 ankle surgery	>1 ankle surgery	No ankle surgeries	1 ankle surgery	>1 ankle surgery
Ankle surgeries total, % (n)	81.5 (299)	10.9 (40)	7.6 (28)	79.7 (118)	15.5 (23)	4.7 (7)
With ankle OA, % (n)	38.2 (13)	29.4 (10)	32.4 (11)	42.9 (3)	42.9 (3)	14.3 (1)
Without ankle OA, % (n)	85.9 (286)	9 (30)	5.1 (17)	81.6 (115)	14.2 (20)	4.3 (6)

*The sum of patients in the subcategories ≤40 years and >40 years does not equal the ‘total number of participants’ due to missing data (regarding age).

n, number; OA, osteoarthritis.

**Table 3:** Association (OR and 95% CI) of severe ankle injury and ankle surgery with ankle osteoarthritis among recently retired professional football and rugby players

	Football (n=401)		Rugby (n=152)	
	Unadjusted	Adjusted*	Unadjusted	Adjusted*
Severe ankle injury, OR (95% CI)	1.34 (1.17 to 1.53)	1.33 (1.14 to 1.55)	1.01 (0.74 to 1.38)	1.05 (0.75 to 1.47)
Ankle surgery, OR (95% CI)	2.20 (1.61 to 3.00)	2.09 (1.41 to 3.08)	1.99 (0.92 to 4.3)	1.96 (0.81 to 4.71)

*Adjusted for age and body mass index.

The prevalence of ankle OA in former professional rugby players was 4.6% (n=7). Overall, six out of seven (86%) rugby players with ankle OA reported to have sustained ≥1 ankle injury, and four out of seven (57.1%) had undergone ≥1 ankle surgery. In the non-OA group (n=145), 58.2% had sustained ≥1 ankle injury and 18.4% had undergone ≥1 ankle surgery. Logistic regression analysis (table 3) showed no significant association between either severe ankle injuries (OR=1.1, 95% CI 0.8 to 1.5) or ankle surgeries (OR=2.0, 95% CI 0.8 to 4.7) and ankle OA among former professional rugby players.

### Mental and physical QoL

For the PROMIS physical health score, a statistically significant lower outcome was found for football (p=0.01) and rugby (p=0.009) players with OA (48 and 45 points, respectively) compared with players without OA (51 and 51 points, respectively). The PROMIS mental health score was not significantly different between players with OA and players without OA. Data are presented in table 4.

**Table 4:** Mental and physical quality of life (0–100) of recently retired football and rugby players with ankle OA compared with players without ankle OA

	Football				Rugby			
	Total (n=401)	With ankle OA (n=37)	Without ankle OA (n=364)	P value	Total (n=152)	With ankle OA (n=7)	Without ankle OA (n=145)	P value
PROMIS mental health (median; IQR; min–max)	51; 46–56; 31–68	51; 46–53; 31–63	53; 46–56; 31–68	0.251	52; 46–56; 28–68	48; 48–53; 46–56	53; 46–56; 28–68	0.567
PROMIS physical health* (median; IQR; min–max)	51; 46–56; 27–68	48; 42–54; 35–62*	51; 48–58; 27–68*	0.01	51; 45–54; 30–68	45; 37–48; 37–51*	51; 37–56; 30–68*	0.009

*Statistically significant difference.

max, maximum; min, minimum; OA, osteoarthritis; PROMIS, Patient-Reported Outcomes Measurement Information System.

### Post-hoc analysis

In the literature, forced retirement is a risk factor for postcareer mental health symptoms.^26^ Following data analysis, we were concerned by the seemingly high percentage of football and rugby players who stopped due to a career-ending injury. As we were analysing the impact ankle OA had on QoL, we felt the effect of a career-ending injury on QoL to be in line with our initial objectives. Consequently we performed a post-hoc analysis to determine any associations between reported forced retirement and mental and physical PROMIS health scores. In this study, 31% of players reported to have been forced to retire, specifically 29% of football players and 36% of rugby players.

The post-hoc subanalysis of all players regarding mental and physical health scores associated with voluntary retirement and in conjunction with ankle OA is presented in table 5. No post-hoc subanalysis was done for football and rugby players separately, regarding voluntary retirement in conjunction with ankle OA, due to the low number of ankle OA cases among rugby players. However mental and physical health scores in football and rugby due to forced retirement are presented separately in table 5.

**Table 5:** Post-hoc analysis regarding mental and physical quality of life of recently retired football and rugby players who reported to have been forced to retire, and quality of life of players who reported to have been forced to retire with ankle OA compared with players without ankle OA

	PROMIS mental health (median; IQR; min–max)	P value	PROMIS physical health (median; IQR; min–max)	P value
Total				
Voluntarily retired* (n=369)	53; 48–56; 31–68*	<0.001	54; 48–58; 30–68*	<0.001
Forced to retire* (n=168)	48; 44–53; 28–68*		48; 42–51; 27–68*	
Voluntarily retired with OA* (n=30)	51; 46–53; 36–63	0.074	51; 45–54; 35–62*	0.001
Voluntarily retired without OA* (n=339)	53; 48–59; 31–68		54; 48–58; 30–68*	
Forced to retire with OA (n=14)	48; 46–55; 31–63	0.841	42; 40–46; 37–58	0.159
Forced to retire without OA (n=154)	48; 44–53; 28–68		48; 42–52; 27–68	
Football*				
Voluntarily retired* (n=272)	53; 48–56; 31–68*	<0.001	54; 48–58; 30–68*	<0.001
Forced to retire* (n=113)	48; 44–53; 31–63*		48; 42–51; 27–62*	
Rugby*				
Voluntarily retired* (n=97)	53; 48–56; 31–68*	0.018	54; 48–58; 35–68*	<0.001
Forced to retire* (n=55)	48; 44–56; 28–68*		48; 37–54; 30–68*	

*Statistically significant difference.

max, maximum; min, minimum; n, number; OA, osteoarthritis; PROMIS, Patient-Reported Outcomes Measurement Information System.

## Discussion

Our most important findings were that (1) the prevalence of ankle OA among former professional football and rugby players was 9.2% (95% CI 6.4 to 12.1) and 4.6% (95% Cl 1.2 to 8.0), respectively; (2) previous severe ankle injuries and ankle surgeries are associated with ankle OA among former professional football players; and (3) ankle OA does not lead to reduced mental or physical QoL in former professional football and rugby players.

### Ankle OA in football and rugby compared with other sports and the general population

Several studies have reported symptomatic ankle OA prevalence among a variety of former elite athletes, ranging from 0% in volleyball (n=22), long distance running (n=30), elite high jump (n=30) and ballet (n=27), to 10% among former military parachutists (n=40).^27–31^ In our study, the prevalence of ankle OA among former professional football (9.2%) and rugby (4.6%) players was higher than the estimated prevalence among the general population (3.4 %).^12^ Murray *et al*^12^ invited participants with self-reported ankle pain (11.7%) from the general population for a radiograph (radiological atlas of foot OA).^32^

Our study reports lower ankle OA prevalence compared with previous studies among former professional football players (12%–19%).^13–15^ In these studies, data were similarly collected using cross-sectional, retrospective, self-reported questionnaires.^13 14^ No previous data are available concerning ankle OA among former professional rugby players. The low ankle OA prevalence among rugby players in this study is likely due to either an under-reporting of ankle OA among rugby players or a type II error.

### Association of severe ankle injuries and ankle surgeries with ankle OA

In our study, football players were 1.3 (95% CI 1.1 to 1.6) and 2.1 (95% CI 1.4 to 3.1) times more likely to develop ankle OA for every ankle injury and ankle surgery, respectively. This is in line with previous studies that showed an association between ankle OA and ankle injuries, stating traumatic ankle injuries to be the main cause of ankle OA (70%–78%).^6 7^ Consequences of ankle injuries are thought to be due to either direct damage to the articular surfaces or change in ankle joint biomechanics (eg, through chronic ankle instability due to ankle ligament damage).^33^ No association was seen for severe ankle injuries or ankle surgeries and ankle OA among rugby players. Although the best estimate for the association of ankle surgeries and OA is similar to football (OR=2), the 95% CI includes 1 (0.8–4.7). This may be due to insufficient power among rugby players, as only seven reported ankle OA.

### Health-related QoL

Filbay *et al*^34^ reported factors such as age, type of sport (contact/collision sports), OA and involuntary retirement from sport to possibly negatively impact QoL.^26 35 36^ However the positive impact associated with elite sport participation, such as pride of accomplishments, social network (through sport participation), and coping and adjustment to musculoskeletal pain (due to resilience and determination), may persist and even compensate reduced physical QoL beyond retirement of the player’s athletic career.^34^

Our post-hoc subanalysis revealed 29% of football players and 36% of rugby players were forced to retire. Although we reported several instances of statistically significant differences in our study for both physical and mental PROMIS health scores, all were similar to the estimated norm for the general population (50 points). Unfortunately, there is no robust minimally clinically important difference threshold for the PROMIS-10 global short form applicable to this population. Consequently, it is difficult to interpret the results. Nevertheless, although statistically significant, the PROMIS mental and physical health scores observed, for both football and rugby players, are unlikely clinically significant. Based on our data, ankle OA does not lead to reduced mental or physical QoL in former professional football and rugby players.

### Strengths and limitations

We had an overall good response rate (56%) and a large group of participating football players (n=401). However, several limitations should be mentioned. First, this study had a cross-sectional design; therefore, no causal association can be determined. Second, there were only seven rugby players with OA, implying a potential serious type II error. Third, recruitment procedures were blinded to the research team, and a non-response analysis could not be performed. Fourth, research-based (retrospective) questionnaires are dependent on the accuracy and understanding of the participant. Professional athletes might generally remember the number of severe ankle injuries or surgeries resulting in a training or match absence of ≥4 weeks. However, we cannot exclude that participants were not able to precisely recall all their sustained severe ankle injuries and ankle surgeries or that they did not consult their most recent medical professional. Finally, our definition of OA did not include a clinical and radiological evaluation.

### Implications for practice

The epidemiological data from this study regarding ankle OA prevalence among former professional football and rugby players, and its association with both severe ankle injuries and ankle surgeries, are an essential part of protecting athletes’ health. In both professional football and rugby, a higher prevalence of ankle OA can be expected compared with the general population. Furthermore, as the majority are post-traumatic, preventive measures and adequate rehabilitation are recommended. Awareness should be raised regarding the increased risk of developing ankle OA following each ankle injury and surgery. Additionally primary and secondary prevention programmes should be implemented. Exercise-based prevention intervention programmes, such as the FIFA 11+ in football and the movement control injury prevention programme in rugby, have found reduced ankle injury incidence and burden.^37 38^ Finally, management of ankle OA is key for retired players, focusing on healthy lifestyle including physical activity, pain alleviation, function and minimising disability.^39–41^ With this intention, FIFPRO has developed an ‘After Career Consultation’.^42^ Future studies will evaluate its relevance.

## Conclusion

This cross-sectional study found an athlete-reported ankle OA prevalence of 9.2% and 4.6% among recently retired professional football and rugby players, respectively. Severe ankle injuries and ankle surgeries were associated with a higher prevalence of ankle OA in former professional football players. No clinically relevant differences in QoL were reported in players with OA compared with players without OA. As a higher prevalence of ankle OA can be expected in both professional football and rugby compared with the general population, and the majority are post-traumatic, primary and secondary preventive measures for ankle injuries and an optimal diagnosis and treatment plan are recommended.

## Supplementary Material

Reviewer comments

Author's manuscript

## References

[1] Sankey RA, Brooks JHM, Kemp SPT, et al The epidemiology of ankle injuries in professional rugby union players. Am J Sports Med 2008;36:2415–24. 10.1177/0363546508322889 18779364

[2] Waldén M, Hägglund M, Ekstrand J Time-trends and circumstances surrounding ankle injuries in men’s professional football: An 11-year follow-up of the UEFA champions league injury study. Br J Sports Med 2013. 10.1136/bjsports-2013-092223 23813486

[3] Fuller CW, Taylor A, Kemp SPT, et al Rugby world cup 2015: world rugby injury surveillance study. Br J Sports Med 2017;51:51–7. 10.1136/bjsports-2016-096275 27461882

[4] Ekstrand J, Krutsch W, Spreco A, et al Time before return to play for the most common injuries in professional football: a 16-year follow-up of the UEFA elite Club injury study. Br J Sports Med 2020;54:421–6. 10.1136/bjsports-2019-100666 31182429 PMC7146935

[5] Williams S, Trewartha G, Kemp S, et al A meta-analysis of injuries in senior men’s professional Rugby Union. Sport Med 2013. 10.1007/s40279-013-0078-1 23839770

[6] Valderrabano V, Horisberger M, Russell I, et al Etiology of ankle osteoarthritis. Clin Orthop Relat Res 2009;467:1800–6. 10.1007/s11999-008-0543-6 18830791 PMC2690733

[7] Saltzman CL, Salamon ML, Blanchard GM, et al Epidemiology of ankle arthritis: report of a consecutive series of 639 patients from a tertiary orthopaedic center. Iowa Orthop J 2005;25:44–6. 16089071 PMC1888779

[8] Drawer S, Fuller CW Evaluating the level of injury in english professional football using a risk based assessment process. Br J Sports Med 2002;36:446–51. 10.1136/bjsm.36.6.446 12453840 PMC1724558

[9] Bathgate A, Best JP, Craig G, et al A prospective study of injuries to elite Australian rugby union players. Br J Sports Med 2002;36:265–9. 10.1136/bjsm.36.4.265 12145116 PMC1724535

[10] Garraway WM, Lee AJ, Hutton SJ, et al Impact of professionalism on injuries in rugby union. Br J Sports Med 2000;34:348–51. 10.1136/bjsm.34.5.348 11049144 PMC1756233

[11] Brooks JHM, Fuller CW, Kemp SPT, et al Epidemiology of injuries in english professional rugby union: part 1 match injuries. Br J Sports Med 2005;39:757–66. 10.1136/bjsm.2005.018135 16183774 PMC1725032

[12] Murray C, Marshall M, Rathod T, et al Population prevalence and distribution of ankle pain and symptomatic radiographic ankle osteoarthritis in community dwelling older adults: a systematic review and cross-sectional study. PLoS One 2018;13:e0193662. 10.1371/journal.pone.0193662 29708977 PMC5927448

[13] Drawer S, Fuller CW Propensity for osteoarthritis and lower limb joint pain in retired professional soccer players. Br J Sports Med 2001;35:402–8. 10.1136/bjsm.35.6.402 11726474 PMC1724418

[14] Turner AP, Barlow JH, Heathcote-Elliott C Long term health impact of playing professional football in the United Kingdom. Br J Sports Med 2000;34:332–6. 10.1136/bjsm.34.5.332 11049141 PMC1756230

[15] Kuijt M-TK, Inklaar H, Gouttebarge V, et al Knee and ankle osteoarthritis in former elite soccer players: a systematic review of the recent literature. J Sci Med Sport 2012;15:480–7. 10.1016/j.jsams.2012.02.008 22572082

[16] Davies MAM, D Judge A, Delmestri A, et al Health amongst former rugby union players: a cross-sectional study of morbidity and health-related quality of life. Sci Rep 2017;7:11786. 10.1038/s41598-017-12130-y 28959048 PMC5620077

[17] Schuring N, Aoki H, Gray J, et al Osteoarthritis is associated with symptoms of common mental disorders among former elite athletes. Knee Surg Sports Traumatol Arthrosc 2017;25:3179–85. 10.1007/s00167-016-4255-2 27488101 PMC5603643

[18] Gouttebarge V, Inklaar H, Frings-Dresen MH Risk and consequences of osteoarthritis after a professional football career: a systematic review of the recent literature. J Sports Med Phys Fitness 2014;54:494–504. 25034551

[19] Vandenbroucke JP, von Elm E, Altman DG, et al Strengthening the reporting of observational studies in epidemiology (STROBE): explanation and elaboration. Int J Surg 2014. 10.1097/EDE.0b013e3181577511 18049195

[20] Woodward M Epidemiology: study design and data analysis. Third ed, 2013.

[21] National Institute for Health and Care Excellence National clinical guideline centre osteoarthritis care and management in adults 2014:1–505. 25340227

[22] Hays RD, Bjorner JB, Revicki DA, et al Development of physical and mental health summary scores from the patient-reported outcomes measurement information system (PROMIS) global items. Qual Life Res 2009;18:873–80. 10.1007/s11136-009-9496-9 19543809 PMC2724630

[23] Song K, Wikstrom EA, Tennant JN, et al Osteoarthritis prevalence in retired National football League players with a history of ankle injuries and surgery. J Athl Train 2019;54:1165–70. 10.4085/1062-6050-421-18 31553650 PMC6863693

[24] Felson DT An update on the pathogenesis and epidemiology of osteoarthritis. Radiol Clin North Am 2004;42:1–9. 10.1016/S0033-8389(03)00161-1 15049520

[25] Hunter DJ, McDougall JJ, Keefe FJ The symptoms of osteoarthritis and the genesis of pain. Med Clin North Am 2009;93:83–100. 10.1016/j.mcna.2008.08.008 19059023

[26] Gouttebarge V, Castaldelli-Maia JM, Gorczynski P, et al Occurrence of mental health symptoms and disorders in current and former elite athletes: a systematic review and meta-analysis. Br J Sports Med 2019;53:700–6. 10.1136/bjsports-2019-100671 31097451 PMC6579497

[27] Gross P, Marti B Risk of degenerative ankle joint disease in volleyball players: study of former elite athletes. Int J Sports Med 1999;20:58–63. 10.1055/s-2007-971094 10090465

[28] Konradsen L, Hansen EM, Søndergaard L Long distance running and osteoarthrosis. Am J Sports Med 1990;18:379–81. 10.1177/036354659001800408 2403186

[29] Schmitt H, Lemke JM, Brocai DRC, et al Degenerative changes in the ankle in former elite high jumpers. Clin J Sport Med 2003;13:6–10. 10.1097/00042752-200301000-00002 12544157

[30] van Dijk CN, Lim LS, Poortman A, et al Degenerative joint disease in female ballet dancers. Am J Sports Med 1995;23:295–300. 10.1177/036354659502300307 7661255

[31] Murray-Leslie CF, Lintott DJ, Wright V The knees and Ankles in sport and veteran military parachutists. Ann Rheum Dis 1977;36:327–31. 10.1136/ard.36.4.327 409358 PMC1006693

[32] Menz HB, Munteanu SE, Landorf KB, et al Radiographic classification of osteoarthritis in commonly affected joints of the foot. Osteoarthritis Cartilage 2007;15:1333–8. 10.1016/j.joca.2007.05.007 17625925

[33] Wikstrom EA, Hubbard-Turner T, McKeon PO Understanding and treating lateral ankle sprains and their consequences: a constraints-based approach. Sports Med 2013;43:385–93. 10.1007/s40279-013-0043-z 23580392

[34] Filbay S, Pandya T, Thomas B, et al Quality of life and life satisfaction in former athletes: a systematic review and meta-analysis. Sports Med 2019;49:1723–38. 10.1007/s40279-019-01163-0 31429036 PMC6789047

[35] Arnold R, Fletcher D A research synthesis and taxonomic classification of the organizational stressors encountered by sport Performers. J Sport Exerc Psychol 2016. 10.1123/jsep.34.3.397 22691400

[36] Wylleman P, Reints A A lifespan perspective on the career of talented and elite athletes: perspectives on high-intensity sports. Scand J Med Sci Sports 2010;20 Suppl 2:88–94. 10.1111/j.1600-0838.2010.01194.x 20840566

[37] Nouni-Garcia R, Carratala-Munuera C, Orozco-Beltran D, et al Clinical benefit of the FIFA 11 programme for the prevention of hamstring and lateral ankle ligament injuries among amateur soccer players. Inj Prev 2018;24:149–54. 10.1136/injuryprev-2016-042267 28642247

[38] Attwood MJ, Roberts SP, Trewartha G, et al Efficacy of a movement control injury prevention programme in adult men’s community rugby union: a cluster randomised controlled trial. Br J Sports Med 2018. 10.1136/bjsports-2017-098005 PMC5867421 29055883

[39] Kirkendall DT, Garrett WE Management of the retired athlete with osteoarthritis of the knee. Cartilage 2012;3:69S–76. 10.1177/1947603511408287 26069611 PMC4297174

[40] McAlindon TE, Bannuru RR, Sullivan MC, et al OARSI guidelines for the non-surgical management of knee osteoarthritis. Osteoarthr Cartil 2014. 10.1016/j.joca.2014.01.003 24462672

[41] Paterson KL, Gates L Clinical assessment and management of foot and ankle osteoarthritis: a review of current evidence and focus on pharmacological treatment. Drugs Aging 2019;36:203–11. 10.1007/s40266-019-00639-y 30680680

[42] Gouttebarge V, Goedhart E, Kerkhoffs G Empowering the health of retired professional footballers: the systematic development of an after career consultation and its feasibility. BMJ Open Sport Exerc Med 2018;4:e000466. 10.1136/bmjsem-2018-000466 PMC6350730 30774974

