# Supplementary material for: Ankle osteoarthritis and its association with severe ankle injuries, ankle surgeries and health-related quality of life in recently retired professional male football and rugby players: a cross-sectional observational study
Source: BMJ Open. 2020 Jun 21;10(6):e036775. doi: 10.1136/bmjopen-2020-036775 (PMC7310999; doi:10.1136/bmjopen-2020-036775)
Supplement: Author's manuscript [file bmjopen-2020-036775.draft_revisions.pdf]

BMJ Open

BMJ Open is committed to open peer review. As part of this commitment we make the peer review history of every article we publish publicly available.

When an article is published we post the peer reviewers' comments and the authors' responses online. We also post the versions of the paper that were used during peer review. These are the versions that the peer review comments apply to.

The versions of the paper that follow are the versions that were submitted during the peer review process. They are not the versions of record or the final published versions. They should not be cited or distributed as the published version of this manuscript.

BMJ Open is an open access journal and the full, final, typeset and author-corrected version of record of the manuscript is available on our site with no access controls, subscription charges or pay-per-view fees (http://bmjopen.bmj.com).

If you have any questions on BMJ Open's open peer review process please email info.bmjopen@bmj.com

BMJ Open

Ankle osteoarthritis and its association with severe ankle injuries, ankle surgeries and health-related quality of life in recently retired professional male football and rugby players

Journal:	*BMJ Open*
Manuscript ID	bmjopen-2020-036775
Article Type:	Original research
Date Submitted by the Author:	02-Jan-2020
Complete List of Authors:	Paget, Liam; Amsterdam UMC - Locatie AMC, Orthopaedic surgery; Amsterdam Collaboration for Health and Safety in Sports, AMC/VUMC IOC Research Center (ACHSS)<br>Aoki, Haruhito; St. Marianna University School of Medicine; Yokohama City Sports Medical Center<br>Kemp, Simon; Rugby Football Union, Sports Medicine<br>Lambert, Mike; University of Cape Town, UCT/MRC Research Unit for Exercise Science and Sports Medicine<br>Readhead, Clint; South Africa Rugby Union, Medical; University of Cape Town Division of Exercise Science and Sports Medicine<br>Stokes, Keith; University of Bath, Department for Health; Rugby Football Union,<br>Viljoen, Wayne; South African Rugby Union, Medical; University of Cape Town Division of Exercise Science and Sports Medicine<br>Reurink, G; Sports Medicine, OLVG, The Sport Physician Group; Academic Center for Evidence-based Sports medicine (ACES)<br>Tol, J; Academic Center for Evidence-Based Sports medicine (ACES); Aspetar Orthopaedic and Sports Medicine Hospital<br>Kerkhoffs, Gino; Amsterdam UMC - Locatie AMC, Orthopaedic surgery; Amsterdam Collaboration for Health and Safety in Sports (ACHSS)<br>Gouttebarge, Vincent; Amsterdam UMC - Locatie AMC, Orthopaedic surgery; Football Players Worldwide (FIFPRO)
Keywords:	EPIDEMIOLOGY, ORTHOPAEDIC & TRAUMA SURGERY, Foot & ankle < ORTHOPAEDIC & TRAUMA SURGERY, Orthopaedic sports trauma < ORTHOPAEDIC & TRAUMA SURGERY, SPORTS MEDICINE

SCHOLARONE™
Manuscripts

I, the Submitting Author has the right to grant and does grant on behalf of all authors of the Work (as defined in the below author licence), an exclusive licence and/or a non-exclusive licence for contributions from authors who are: i) UK Crown employees; ii) where BMJ has agreed a CC-BY licence shall apply, and/or iii) in accordance with the terms applicable for US Federal Government officers or employees acting as part of their official duties; on a worldwide, perpetual, irrevocable, royalty-free basis to BMJ Publishing Group Ltd ("BMJ") its licensees and where the relevant Journal is co-owned by BMJ to the co-owners of the Journal, to publish the Work in this journal and any other BMJ products and to exploit all rights, as set out in our licence.

The Submitting Author accepts and understands that any supply made under these terms is made by BMJ to the Submitting Author unless you are acting as an employee on behalf of your employer or a postgraduate student of an affiliated institution which is paying any applicable article publishing charge ("APC") for Open Access articles. Where the Submitting Author wishes to make the Work available on an Open Access basis (and intends to pay the relevant APC), the terms of reuse of such Open Access shall be governed by a Creative Commons licence – details of these licences and which Creative Commons licence will apply to this Work are set out in our licence referred to above.

Other than as permitted in any relevant BMJ Author's Self Archiving Policies, I confirm this Work has not been accepted for publication elsewhere, is not being considered for publication elsewhere and does not duplicate material already published. I confirm all authors consent to publication of this Work and authorise the granting of this licence.

For peer review only

Ankle osteoarthritis and its association with severe ankle injuries, ankle surgeries and health-related quality of life in recently retired professional male football and rugby players

Liam D. A. Paget[1,2,3], Haruhito Aoki[4, 5], Simon Kemp[6], Mike Lambert[7], Clint Readhead[7, 8], Keith Stokes[6, 9, 10], Wayne Viljoen[7, 8], Gustaaf Reurink[1,2,3,11], Johannes L. Tol[2,3,12], Gino M. M. J. Kerkhoffs[1,2,3], Vincent Gouttebarge[1,2,3,4,13]

[1] Amsterdam UMC, University of Amsterdam, Department of Orthopaedic Surgery, Amsterdam Movement Sciences, Meibergdreef 9, Amsterdam, the Netherlands

[2] Academic Center for Evidence based Sports medicine (ACES), The Netherlands; Amsterdam

[3] Amsterdam Collaboration on Health & Safety in Sports (ACHSS), AMC/VUmc IOC Research Center of Excellence, Amsterdam, The Netherlands

[4] St. Marianna University School of Medicine, Kawasaki, Japan

[5] Yokohama City Sports Medical Center, Yokohama, Japan

[6] Rugby Football Union, Twickenham, UK

[7] Division of Exercise Science and Sports Medicine (ESSM), University of Cape Town, Cape Town, South Africa

[8] South African Rugby Union (SARU), Cape Town, South Africa

[9] Department for Health, University of Bath, Bath, UK

[10] Centre for Sport, Exercise and Osteoarthritis Research Versus Arthritis

[11] The Sport Physician Group, Department of Sports Medicine, OLVG, Amsterdam, The Netherlands

[12] Aspetar, Orthopaedic and Sports Medicine Hospital, Doha, Qatar

[13] Football Players Worldwide (FIFPRO), Hoofddorp, The Netherlands

For peer review only

For peer review only - http://bmjopen.bmj.com/site/about/guidelines.xhtml

Abstract

Objectives

To determine (i) the prevalence of ankle osteoarthritis (OA) among former professional football and rugby players, (ii) assess the association between ankle injuries or ankle surgeries with ankle OA, and (iii) compare the mental and physical quality of life (QoL) between former professional football and rugby players with and without OA.

Methods

We conducted a questionnaire based observational study with a cross-sectional design. Former professional football and rugby players were recruited by the football players worldwide (FIFPRO) and the International Rugby Players (IRP). Information concerning ankle OA, sustained ankle injuries and ankle surgeries was gathered (medical record or team doctor). Health related QoL was assessed using the PROMIS physical and mental health scores.

Results

Overall, 553 former professional football (n = 401) and rugby players (n = 152) were enrolled in the study (response rate of 56%). Ankle OA prevalence among former professional football and rugby players was 9.2% and 4.6%, respectively. Football players were more likely to suffer from ankle OA following every ankle injury and/or surgery. Football and rugby players with ankle OA had similar PROMIS physical and mental health scores to the norm for the general population.

Conclusion

Former professional football and rugby players had a higher ankle OA prevalence than the general population (3.4%). Football players are more likely to suffer from ankle OA following every ankle injury

For peer review only - http://bmjopen.bmj.com/site/about/guidelines.xhtml

and/or surgery. No clinical relevant difference was seen for physical or mental health related QoL among football and rugby players. Preventive measures for ankle injuries are recommended.

Strengths and limitations

- Overall good response rate (56%) and a large group of participating football players (401)
- This study had a cross-sectional design, therefore no causal-association can be determined
- There were only 7 rugby players with OA, implying a potential serious type II error.
- Research based (retrospective) questionnaires are dependent on the accuracy and understanding of the participant. Professional athletes might generally remember the number of severe ankle injuries or surgeries resulting in a training or match absence of ≥4 weeks. However, we cannot exclude that participants were not able to precisely recall all their sustained severe ankle injuries and ankle surgeries or that they did not consult their last (team) doctor
- Our OA definition did not include a clinical and radiological evaluation.

For peer review only

Background

Football and Rugby Union (hereafter 'rugby') are among the most popular team sports worldwide. In both sports, ankle injuries represent a significant proportion of the total injuries sustained.[1–5] Previous ankle injuries are an important risk factor for ankle osteoarthritis (OA).[6,7]

Drawer et al. (2002) estimates the overall risk of injury in professional football to be 1,000 times higher than in high-risk industrial occupations, with ankle injuries contributing 10-18% of all injuries.[2,8] The Union of European Football Associations (UEFA)Champions League Injury Study reported an overall ankle injury rate of 1 injury per 1000 hours.[2] Lateral ankle sprains (51%) were the most prevalent sub-type of ankle injuries.[2] In professional rugby, epidemiological studies show that ankle injuries contribute 5-20% of all injuries.[1,3,9,10] Similar to professional football, lateral ankle sprains are the most common ankle injuries with an injury rate of 3.8 - 4.5 injuries per 1,000 player hours depending on the position on the field.[11]

Two retrospective cohort studies from the general population (n = 390 and n = 639 patients respectively), reported that 70-78% of ankle OA cases were associated with a previously sustained ankle injury. These previous injuries included fractures, isolated osteochondral defects of the talus and ankle ligament injuries.[6,7] Among these post-traumatic ankle OA cases, 28% were associated with a previously sustained ligamentous injury.[7] To our knowledge, there are no prospective studies on the association of ankle injuries and ankle OA.

In the general population the prevalence of ankle OA is estimated to be 3.4% in people over 50 years of age. [12] The reported prevalence of ankle OA 16 to 22 years after a professional football career is 12-19%.[13–15] No data are available for ankle OA among former professional rugby players. In comparison to

For peer review only - http://bmjopen.bmj.com/site/about/guidelines.xhtml

a general population control group, the prevalence of OA in any joint and the incidence of joint replacement for any joint was estimated to be 4 and 6 times higher, respectively, among former elite rugby players.[16] This has been associated with a significantly poorer mental and physical health-related quality of life (QoL).[16]

Several other studies report the association of general OA with mental health symptoms and physical health-related QoL among former elite athletes across professional team sports including football and rugby.[17,18] Therefore, the impact of OA on retired players must be considered. To date, the epidemiological evidence concerning the prevalence of ankle OA in professional football is either outdated or limited, and in the case of rugby non-existent, while data on the association between previous ankle injury and ankle OA is scarce in both sports.

The objective of this study was threefold. Firstly, to evaluate the prevalence of ankle OA in recently retired professional football and rugby players. Secondly, to determine the association between ankle injury and/or ankle surgery and ankle OA. Thirdly, to compare the mental and physical QoL in players with and without ankle OA. Our hypotheses were that (i) recently retired professional football and rugby players have a higher ankle OA prevalence than the general population (as found in the literature), (ii) ankle injuries and/or ankle surgeries are associated with ankle OA, and (iii) the mental and physical QoL of recently retired professional football and rugby players with OA is lower compared to those players without OA.

For peer review only - http://bmjopen.bmj.com/site/about/guidelines.xhtml

109 **Methods**

110 ***Design***

111 We conducted a questionnaire based observational study with a cross-sectional design. Ethical approval
112 for the study was provided by the Ethical Committee of the Yokohama City Sports Medical Center (17.003;
113 Yokohama, Japan) and the Medical Ethics Review Committee of the Academic Medical Center
114 (W16_366#16.431; Amsterdam, The Netherlands). The study was reported according to the
115 'Strengthening the Reporting of Observational Studies in Epidemiology' statement and conducted in
116 accordance with the Declaration of Helsinki (2013).[19]

117

118 ***Participants***

119 Retired professional football and rugby players were randomly recruited to reduce recruitment bias, by
120 the Football Players Worldwide (FIFPRO) and the International Rugby Players (IRP). Inclusion criteria were:
121 (a) being a retired male professional football or rugby player; (b) being younger than 50 years of age; (c)
122 being able to read and comprehend texts in English, French or Spanish.

123

124 The definition of a retired professional football or rugby player was that he (i) has trained to improve
125 performances, (ii) has competed in the highest or second highest national league, and (iii), has had training
126 and competition as a major activity (way of living) or focus of personal interest, devoting several hours in
127 all or most of the days for these activities, and exceeding the time allocated to other types of professional
128 or leisure activities. If interested, players received information about the study, gave their informed
129 consent and completed an electronic questionnaire. Once completed, the electronic questionnaires were
130 saved automatically on a secured electronic server that only the principal investigator could access.

131

132 Sample size calculation for ankle OA prevalence indicated that 138 participants per sport were needed

For peer review only - http://bmjopen.bmj.com/site/about/guidelines.xhtml

(power of 80%, confidence interval of 95%; absolute precision of 5%) under the assumption of an anticipated population proportion of 10%.[20] Expecting a response rate of approximately 50%, we intended to reach at least 300 participants per sport.

Dependent variable: Ankle osteoarthritis

The clinical diagnosis of ankle OA by a medical professional was retrospectively determined using a single question ('Have you been diagnosed with ankle osteoarthritis by a medical professional?'). Participants were given the definition of ankle OA (based on the NICE criteria; adapted for age), determined as the damage of the ankle joint's cartilage that leads to activity-related joint pain with either no morning joint-related stiffness or morning stiffness that lasts no longer than 30 minutes.[21] For this question, participants were requested to consult either their medical record or their most recent medical professional.

Independent variable: Severe ankle injury and related surgery

History of a severe ankle injury during their professional football or rugby career was examined through a single question for the sequential recording of single or multiple injury events over time. Similarly, history of an ankle related surgery was recorded through a single question for the sequential recording of a single or multiple ankle surgeries over time. In our study, a severe ankle injury was defined as an injury that involved the ankle joint, occurred during team activities (training or match), and led to absence from either training or a match for more than 28 days [18,19]. For this question, participants were requested to consult either their medical records or their most recent medical doctor.

Health-related quality of life

The Patient-Reported Outcomes Measurement Information System Global Health short form (PROMIS-GH) assesses multiple domains related to health-related quality of life such as health, functioning, pain,

For peer review only - http://bmjopen.bmj.com/site/about/guidelines.xhtml

social activities and fatigue.[20] The PROMIS-GH has been validated in several populations and languages among which are English, French and Spanish (for detailed information, see www.nihpromis.org).[20] The Global Mental Health and Global Physical Health scores were calculated based on 10 items each measured on a 5-point scale (from 1 to 5) and subsequently converted.[20] These subscale scores ranged from 0 to 100, with a higher score indicating better quality of life and a mean score of 50 indicating the norm for the general population.[22]

Procedures

An anonymous questionnaire (electronic and/or paper) available in English, French and Spanish was compiled (LimeSurvey Professional). It included the following descriptive variables: age, body-height, body-weight, duration of professional football or rugby career, level of play, duration and nature of retirement, and current employment status (employed or not employed). Additionally, this questionnaire included questions for the outcomes previously elaborated on (ankle OA, ankle injury, ankle surgery, QoL). Information about the study was sent per email to potential participants by FIFPRO and IRP. Participants interested in the study, gave their informed consent and completed the electronic questionnaire. Participants were asked to complete the questionnaire within 2 weeks, reminders being sent after 2 and 4 weeks. For privacy reasons, the responses to the questionnaires were coded and anonymized. Once completed, the electronic questionnaires were automatically saved on a secured electronic server that only the principal investigator could access. Players participated voluntarily and did not receive any reward for their participation.

Statistical analyses

Data analysis was performed using the statistical software IBM SPSS 24.0 for Windows. Analyses were conducted separately for retired professional football and rugby players. Data were assessed (visually) for

For peer review only - http://bmjopen.bmj.com/site/about/guidelines.xhtml

normality and presented as mean ± standard deviation (SD), median (interquartile range [IQR]), or frequency (proportion in %) as appropriate. Body mass index (BMI) was calculated using the provided weight and height of participants (kg/m^2). Prevalence of ankle OA, overall and within the age categories of ≤40 years and >40 years, was calculated as the proportion of the number of participants with ankle OA relative to the total number of participants in each category.[20] A logistic regression analysis was performed to determine the association of the number of severe ankle injuries and/or ankle surgeries (continuous independent variable) with ankle OA (dichotomous dependent variable). Severe ankle injuries and or ankle-related surgeries were also expressed using 3 pre-determined categories (0, 1 and >1). This was adjusted for age and BMI, both having been identified as risk factors for OA.[23,24] Descriptive analyses of health-related quality of life (Global Physical Health and Global Mental Health) were conducted, while comparisons between groups (retired players with ankle OA vs. retired players without ankle OA) were made using a Mann-Whitney U test for independent samples.[20]

For peer review only - http://bmjopen.bmj.com/site/about/guidelines.xhtml

Results

Participants

From a total of 750 football and 326 rugby players contacted, respectively 401 and 152 gave their written informed consent and completed the questionnaire (overall response rate of 56%). Characteristics of the two groups including the pre-determined sub-categories for severe ankle injuries and ankle surgeries (0, 1 and >1) are illustrated in Table 1.

Prevalence of ankle osteoarthritis, severe injury and surgery

The prevalence of patient reported ankle OA (including the pre-determined categories of ≤ 40 years and > 40 years) among recently retired professional football and rugby players, is presented in Table 2. The prevalence of ankle OA in former professional football players was 9.2% (n = 37). Overall, 94% of players with ankle OA were documented to have sustained ≥1 ankle injury and 62% had undergone ≥1 ankle surgery. In the non-OA group (n = 364), 54% had sustained ≥1 ankle injury and 14% had undergone ≥1 ankle surgery. Logistic regression analysis (Table 3) showed that football players who had sustained a severe ankle injury were 1.3 (95%CI: 1.1 - 1.6) times more likely to report ankle OA (p < 0.001). Football players who had undergone ankle surgery were 2.1 (95%CI: 1.4 – 3.1) times more likely to report ankle OA (p< 0.001).

The prevalence of ankle OA in former professional rugby players was 4.6% (n = 7). Overall, six out of seven (86%) rugby players with ankle OA reported to have sustained ≥1 ankle injury and four out of seven (57.1%) had undergone ≥1 ankle surgery. In the non-OA group (n = 145), 58.2% had sustained ≥1 ankle injury and 18.4% had undergone ≥1 ankle surgery. Logistic regression analysis (Table 3) showed no significant association between either severe ankle injuries (OR = 1.1 95%CI: 0.8 – 1.5) or ankle surgeries (OR = 2.0 95%CI: 0.8 – 4.7) and ankle OA among former professional rugby players.

For peer review only - http://bmjopen.bmj.com/site/about/guidelines.xhtml

Table 1: Descriptive characteristics of recently retired professional football players and rugby players

	Football			Rugby		
	Total (n = 401)	Ankle OA (n = 37)	No ankle OA (n = 364)	Total (n = 152)	Ankle OA (n = 7)	No ankle OA (n = 145)
*Age in years * (median; IQR; min-max / mean ± SD)*	36 (32-40; 25-50)	39 (36-42; 27-49)*	36 (32-40; 25-50)*	40 ± 6	41 ± 7	40 ± 6
Height in cm (mean ± SD / median; IQR; min-max)	181 ± 7	180 ± 7	182 ± 7	186 (180-191; 166-203)	191 (173-192; 172-192)	186 (180-191; 166-203)
Weight in kg (in kg (mean ± SD))	82 ± 9	82 ± 13	82 ± 9	100 (91-114; 59-170)	108 (89-123; 82-130)	100 (91-113; 59-170)
BMI in kg/m² (median; IQR; min-max))	24.8 (23.5-26.2; 20.1-32.7)	24.8 (22.9-26.6; 20.3-29)	24.7 (23.5-26.1; 20.1-32.7)	29.0 (27.1-31.4; 20.4-52.5)	32.2 (28.5-33.7; 27.4-35.3)*	28.9 (27.0-31.2; 20.4-52.5)*
Employed in % (n)	89% (350)	92% (33)	89% (317)	91% (139)	86% (6)	92% (133)
Professional career duration in years (median; IQR; min-max)	13 (10-15; 2-19)	13 (12-16; 5-18)	13 (9-15; 2-19)	10 (7-15; 2-20)	10 (8-12.5; 8-13)	10 (7-15; 2-20)
Retirement duration in years (median; IQR; min-max)	4 (2-7; 1-17)	5 (3-9; 1-12)	4 (2-7; 1-17)	8 (3-12; 0-22)	12 (2-16; 1-19)	8 (4-11; 0-22)
Level of play top league; % (n)	81% (314)	92% (34)	80% (280)	80% (106)	86% (6)	80% (100)
Forced retirement % (n)	29% (113)	27% (10)	30% (103)	36% (55)	57% (4)	35% (51)
Severe ankle injuries (median; IQR; min-max)	1 (0-2; 0-10)	2 (1-3; 0-10)	1 (0-2; 0-10)	1 (0-2; 0-20)	1 (1-2; 0-3)	1 (0-2; 0-20)
Ankle surgeries (median; IQR; min-max)	0 (0-0; 0-5)	1 (0-2; 0-5)	0 (0-0; 0-5)	0 (0-0; 0-4)	1 (0-1; 0-2)	0 (0-0; 0-4)

For peer review only - http://bmjopen.bmj.com/site/about/guidelines.xhtml

* Statistically significant difference between ankle OA and no ankle OA. Abbreviations: OA = Osteoarthritis; IQR = interquartile range; n = number

For peer review only

For peer review only - http://bmjopen.bmj.com/site/about/guidelines.xhtml

Table 2: Subcategories for prevalence of reported ankle OA, severe injuries and ankle surgeries in retired football and rugby players

	**Football**			**Rugby**		
	Total (n = 401)*	≤ 40 years (n = 271)	> 40 years (n = 83)	Total (n = 152)	≤ 40 years (n = 82)	> 40 years (n = 66)
Prevalence ankle OA in % (95%CI)	9.2% (6.4 –12.1)	8.5% (5.2 – 11.8)	12.0% (5.0 – 19.1)	4.6% (1.2 – 8.0)	2.4% (0.9 – 5.8)	4.5% (0.5 – 9.6)
	0 ankle injuries	1 ankle injury	> 1 ankle injury	0 ankle injuries	1 ankle injury	> 1 ankle injury
Severe ankle injuries total (%, (n))	42.5% (156)	25.3% (93)	32.2% (118)	40.5% (60)	27.7% (41)	31.8% (47)
- *With ankle OA (%, (n))*	5.7% (2)	28.6% (10)	65.7% (23)	14.3% (1)	42.9% (3)	42.9% (3)
- *Without ankle OA (%, (n))*	46.4% (154)	25.0% (83)	28.6% (95)	41.8% (59)	27.0% (38)	31.2% (44)
	No ankle surgeries	1 ankle surgery	> 1 ankle surgery	No ankle surgeries	1 ankle surgery	> 1 ankle surgery
Ankle surgeries total in % (n)	81.5% (299)	10.9% (40)	7.6% (28)	79.7% (118)	15.5% (23)	4.7% (7)
- *With ankle OA % (n)*	38.2% (13)	29.4% (10)	32.4% (11)	42.9% (3)	42.9% (3)	14.3% (1)
- *Without ankle OA % (n)*	85.9% (286)	9% (30)	5.1% (17)	81.6% (115)	14.2% (20)	4.3% (6)

**The sum of patients in the subcategories ≤ 40 years and > 40 years does not equal the "Total number of participants" due to missing data (regarding age).*
Abbreviations: OA = Osteoarthritis; n = number; CI = confidence interval;

For peer review only - http://bmjopen.bmj.com/site/about/guidelines.xhtml

Table 3: Association (odds ratio and 95% CI) of severe ankle injury and ankle surgery with ankle osteoarthritis among recently retired professional football and rugby players

	Football (n = 401)		Rugby (n = 152)	
	Unadjusted	Adjusted$^{\psi}$	Unadjusted	Adjusted$^{\psi}$
Severe ankle injury (OR, 95%CI)	1.34 (1.17-1.53)	1.33 (1.14-1.55)	1.01 (0.74-1.38)	1.05 (0.75-1.47)
Ankle surgery (OR, 95%CI)	2.20 (1.61-3.00)	2.09 (1.41-3.08)	1.99 (0.92-4.3)	1.96 (0.81-4.71)

$^{\psi}$Adjusted for age and body mass index (BMI); Abbreviations: OR = Odds Ratio; n = number; CI = confidence interval

For peer review only

Mental and physical QoL

For the PROMIS physical health score a statistically significant lower outcome was found for football (p = 0.01) and rugby players (p = 0.009) with OA (48 and 45 points respectively) compared to players without OA (51 and 51 points respectively). The PROMIS mental health score was not significantly different between players with OA vs players without OA. Data is presented in Table 4.

Table 4: Mental and physical quality of life (0-100) of recently retired football and rugby players with ankle OA compared to players without ankle OA

	**Football**				**Rugby**			
	Total (n = 401)	With ankle OA (n = 37)	Without ankle OA (n = 364)	p-value	Total (n = 152)	With ankle OA (n = 7)	Without ankle OA (n = 145)	p-value
PROMIS mental health (median; IQR; min-max)	51; 46-56; 31-68	51; 46-53; 31-63	53; 46-56; 31-68	0.251	52; 46-56; 28-68	48; 48-53; 46-56	53; 46-56; 28-68	0.567
PROMIS Physical health (median; IQR; min-max)*	51; 46-56; 27-68	48; 42-54; 35-62*	51; 48-58; 27-68*	0.010	51; 45-54; 30-68	45; 37-48; 37-51*	51; 37-56; 30-68*	0.009

**Statistically significant difference;* Abbreviations: OA = Osteoarthritis; IQR = Interquartile range; n = number

For peer review only - http://bmjopen.bmj.com/site/about/guidelines.xhtml

Table 5: Post-Hoc analysis regarding mental and physical quality of life of recently retired football and rugby players who reported to have been forced to retire, and quality of life of players who reported to have been forced to retire with ankle OA compared to players without ankle OA

	**PROMIS mental health** (median; IQR; min-max)	**p-value**	**PROMIS physical health** (median; IQR; min-max)	**p-value**
Total				
Voluntarily retired (n = 369)*	53; 48-56; 31-68*	p < 0.001	54; 48-58; 30-68*	p < 0.001
Forced to retire (n = 168)*	48; 44-53; 28-68*		48; 42-51; 27-68*	
Voluntarily retired with OA (n = 30)*	51; 46-53; 36-63	p = 0.074	51; 45-54; 35-62*	p = 0.001
Voluntarily retired without OA (n = 339)*	53; 48-59; 31-68		54; 48-58; 30-68*	
Forced to retire with OA (n = 14)	48; 46-55; 31-63	p = 0.841	42; 40-46; 37-58	p = 0.159

Post-hoc analysis

In the literature, forced retirement is a risk factor for post-career mental health symptoms.[25] Following data analysis, we were concerned by the seemingly high percentage of football and rugby players who stopped due to a career ending injury. As we were analysing the impact ankle OA had on QoL, we felt the effect of a career ending injury on QoL to be in line with our initial objectives. Consequently we performed a post-hoc analysis to determine any associations between reported forced retirement and mental and physical PROMIS health scores. In this study, 31% of players reported to have been forced to retire, specifically: 29% of football players and 36% of rugby players.

The post-hoc sub-analysis of all players regarding mental and physical health scores associated with voluntary retirement and in conjunction with ankle OA is presented in Table 5. No post-hoc sub-analysis was done for football and rugby players separately, regarding voluntary retirement in conjunction with ankle OA, due to the low number of ankle OA cases among rugby players. However mental and physical health scores in football and rugby due to forced retirement is presented separately in Table 5.

For peer review only

For peer review only - http://bmjopen.bmj.com/site/about/guidelines.xhtml

Forced to retire without OA (n = 154)	48; 44-53; 28-68		48; 42-52; 27-68	
*Football**				
Voluntarily retired (n = 272)*	53; 48-56; 31-68*	p < 0.001	54; 48-58; 30-68*	p < 0.001
Forced to retire (n = 113)*	48; 44-53; 31-63*		48; 42-51; 27-62*	
*Rugby**				
Voluntarily retired (n = 97)*	53; 48-56; 31-68*	p = 0.018	54; 48-58; 35-68*	p < 0.001
Forced to retire (n = 55)*	48; 44-56; 28-68*		48; 37-54; 30-68*	

**Statistically significant difference; Abbreviations: IQR = Interquartile range; OA = Osteoarthritis; n = number*

For peer review only

Discussion

Our most important findings were that (i) the prevalence of ankle OA among former professional football and rugby players was, 9.2% (95%CI 6.4 –12.1) and 4.6% (95%Cl (1.2 – 8.0), respectively; (ii) previous severe ankle injuries and ankle surgeries are associated with ankle OA among former professional football players; and (iii) ankle OA does not lead to reduced mental or physical QoL in former professional football and rugby players.

Ankle OA in football and rugby compared to other sports and the general population

Several studies have reported symptomatic ankle OA prevalence among a variety of former elite athletes, ranging from 0% in volleyball (n = 22), long distance running (n = 30), elite high-jump (n = 30) and ballet (n = 27) to 10% among former military parachutists (n = 40).[26–30] In our study, the prevalence of ankle OA among former professional football (9.2%) and rugby players (4.6%) was higher than the estimated prevalence among the general population (3.4 %).[12] Murray et al. (2018) invited participants with self-reported ankle pain (11.7%) from the general population for a radiograph (radiological atlas of Foot OA).[12,31]

Our study reports a lower ankle OA prevalence compared to previous studies among former professional football players (12-19%).[13–15] In these studies, data was similarly collected using cross-sectional retrospective self-reported questionnaires.[13,14] No previous data is available concerning ankle OA among former professional rugby players. The low ankle OA prevalence among rugby players in this study is likely due to either an under reporting of ankle OA among rugby players or a type II error.

Association of severe ankle injuries and ankle surgeries with ankle OA

In our study, football players were 1.3 (95%CI: 1.1 - 1.6) and 2.1 (95%CI: 1.4 – 3.1) times more likely to develop ankle OA for every ankle injury and ankle surgery, respectively. This is in line with previous

For peer review only

studies that showed an association between ankle OA and ankle injuries, stating traumatic ankle injuries to be the main cause of ankle OA (70-78%).[6,7] Consequences of ankle injuries are thought to be due to either direct damage the articular surfaces or change in ankle joint biomechanics (e.g. through chronic ankle instability due to ankle ligament damage).[32] No association was seen for severe ankle injuries or ankle surgeries and ankle OA among rugby players. Although the best estimate for the association of ankle surgeries and OA is similar to football (OR = 2), the 95%CI includes 1 (0.8 – 4.7). This may be due to insufficient power among rugby players, as only 7 reported ankle OA.

Health-related quality of life

Filbay et al. (2019) reported factors such as age, type of sport (contact/collision sports), OA and involuntary retirement from sport to possibly negatively impact QoL.[25,33–35] However the positive impacts associated with elite sport participation, such as pride of accomplishments, social network (through sport participation) and coping and adjustment to musculoskeletal pain (due to resilience and determination), may persist and even compensate reduced physical QOL beyond retirement of the player's athletic career.[33]

Our post-hoc sub-analysis revealed 29% of football players and 36% of rugby players were forced to retire. Although we reported several instances of statistically significant differences in our study for both physical and mental PROMIS health scores, all were similar to the estimated norm for the general population (50 points). Unfortunately, there is no robust minimally clinically important difference (MCID) threshold for the PROMIS-10 global short form applicable for this population. Consequently, it is difficult to interpret the results. Nevertheless, although statistically significant, the PROMIS mental and physical health scores observed, for both football and rugby players, are unlikely clinically significant.

For peer review only - http://bmjopen.bmj.com/site/about/guidelines.xhtml

Based on our data, ankle OA does not lead to reduced mental or physical QoL in former professional football and rugby players.

Strengths and limitations

We had an overall good response rate (56%) and a large group of participating football players (401). However, several limitations should be mentioned. Firstly, this study had a cross-sectional design, therefore no causal-association can be determined. Secondly, there were only 7 rugby players with OA, implying a potential serious type II error. Thirdly, recruitment procedures were blinded to the research team, a non-response analysis could not be performed. Fourthly, research based (retrospective) questionnaires are dependent on the accuracy and understanding of the participant. Professional athletes might generally remember the number of severe ankle injuries or surgeries resulting in a training or match absence of ≥4 weeks. However, we cannot exclude that participants were not able to precisely recall all their sustained severe ankle injuries and ankle surgeries or that they did not consult their last (team) doctor. Finally, our OA definition did not include a clinical and radiological evaluation.

Implications for practice

The epidemiological data from this study regarding ankle OA prevalence among former professional football and rugby players, and its association with both severe ankle injuries and ankle surgeries, is an essential part of protecting the athlete's health. In both professional football and rugby, a higher prevalence of ankle OA can be expected compared to the general population. Furthermore, As the majority are post-traumatic, preventive measures and adequate rehabilitation are recommended. Awareness should be raised, regarding the increased risk of developing ankle OA following each ankle injury and surgery. Additionally primary and secondary prevention programmes should be implemented. Exercise based prevention intervention programs, such as the FIFA11+ in football and the movement

For peer review only - http://bmjopen.bmj.com/site/about/guidelines.xhtml

control injury prevention programme in rugby, have found reduced ankle injury incidence and burden.[36,37] Finally, management of ankle OA is key for retired players, focusing on healthy lifestyle including physical activity, pain alleviation, function and minimising disability.[38–40] With this intention, FIFPRO has developed an "After Career Consultation".[41] Future studies will evaluate its relevance.

New findings and impact on clinical practice in the future

- The prevalence of ankle OA in both professional football (9.2%) and rugby (4.6%), is higher than the estimated prevalence in the general population (3.4%).
- Awareness should be raised among football and rugby players, regarding the increased risk of developing ankle OA following each ankle injury and ankle surgery.
- Primary and secondary prevention programmes should be implemented, as these might help in reducing the risk of developing ankle OA.
- Management of ankle OA is key for retired players. With this intention, FIFPRO has developed an "After Career Consultation". Future studies will evaluate its relevance.

For peer review only

Conclusion

This cross-sectional study found an athlete reported ankle OA prevalence of 9.2% and 4.6% among recently retired professional football and rugby players, respectively. Severe ankle injuries and ankle surgeries were associated with a higher prevalence of ankle OA in former professional football players. No clinically relevant differences in QoL were reported in players with OA compared to players without OA. As a higher prevalence of ankle OA can be expected in both professional football and rugby

For peer review only - http://bmjopen.bmj.com/site/about/guidelines.xhtml

compared to the general population, and the majority are post-traumatic, primary and secondary preventive measures for ankle injuries and an optimal diagnosis and treatment plan are recommended.

Acknowledgements

The Authors would like to thank all football and ruby players' associations involved. The authors are grateful to all retired professional football and rugby players who participated in the study.

Contributorship statement

Authorship was determined according to the ICMJE authorship recommendations. All authors were involved in the design of the study, data analysis and data interpretation. VG and GMMJK were responsible for data collection. LP drafted the manuscript, with critical review provided by all authors. All authors approved the final version of the manuscript.

Funding

This research received specific grant from any funding agency in the public, commercial or non-profit sectors.

Competing interests

None declared.

Ethics approval

Ethical approval for the study was provided by the Ethical Committee of the Yokohama City Sports Medical Center (17.003; Yokohama, Japan) and the Medical Ethics Review Committee of the Academic Medical Center (W16_366#16.431; Amsterdam, The Netherlands)

For peer review only

Patient and public involvement

This research was done without patient involvement. Patients were not invited to comment on the study design and were not consulted to develop patient relevant outcomes or interpret the results. Patients were not invited to contribute to the writing or editing of this document for readability or accuracy.

Data sharing statement

No additional data available

For peer review only

For peer review only - http://bmjopen.bmj.com/site/about/guidelines.xhtml

References

1. Sankey RA, Brooks JHM, Kemp SPT, Haddad FS. The epidemiology of ankle injuries in professional rugby union players. *Am J Sports Med*. 2008. doi:10.1177/0363546508322889

2. Waldén M, Hägglund M, Ekstrand J. Time-trends and circumstances surrounding ankle injuries in men's professional football: An 11-year follow-up of the UEFA Champions League injury study. *Br J Sports Med*. 2013. doi:10.1136/bjsports-2013-092223

3. Fuller CW, Taylor A, Kemp SPT, Raftery M. Rugby World Cup 2015: World Rugby injury surveillance study. *Br J Sports Med*. 2017;51(1):51-57. doi:10.1136/bjsports-2016-096275

4. Ekstrand J, Krutsch W, Spreco A, et al. Time before return to play for the most common injuries in professional football: a 16-year follow-up of the UEFA Elite Club Injury Study. *Br J Sports Med*. 2019:bjsports-2019-100666. doi:10.1136/bjsports-2019-100666

5. Williams S, Trewartha G, Kemp S, Stokes K. A meta-analysis of injuries in senior men's professional Rugby Union. *Sport Med*. 2013. doi:10.1007/s40279-013-0078-1

6. Valderrabano V, Horisberger M, Russell I, Dougall H, Hintermann B. Etiology of ankle osteoarthritis. *Clin Orthop Relat Res*. 2009. doi:10.1007/s11999-008-0543-6

7. Saltzman CL, Salamon ML, Blanchard GM, et al. Epidemiology of ankle arthritis: report of a consecutive series of 639 patients from a tertiary orthopaedic center. *Iowa Orthop J*. 2005.

8. Drawer S, Fuller CW. Evaluating the level of injury in English professional football using a risk based assessment process. *Br J Sports Med*. 2002. doi:10.1136/bjsm.36.6.446

9. Bathgate A, Best JP, Craig G, Jamieson M. A prospective study of injuries to elite Australian rugby union players. *Br J Sports Med*. 2002. doi:10.1136/bjsm.36.4.265

10. Garraway WM, Lee AJ, Hutton SJ, Russell EBAW, Macleod DAD. Impact of professionalism on injuries in rugby union. *Br J Sports Med*. 2000. doi:10.1136/bjsm.34.5.348

11. Brooks JHM, Fuller CW, Kemp SPT, Reddin DB. Epidemiology of injuries in English professional

For peer review only

For peer review only - http://bmjopen.bmj.com/site/about/guidelines.xhtml

394 rugby union: Part 1 match injuries. *Br J Sports Med*. 2005. doi:10.1136/bjsm.2005.018135
395 12. Murray C, Marshall M, Rathod T, Bowen CJ, Menz HB, Roddy E. Population prevalence and
396 distribution of ankle pain and symptomatic radiographic ankle osteoarthritis in community
397 dwelling older adults: A systematic review and cross-sectional study. *PLoS One*. 2018.
398 doi:10.1371/journal.pone.0193662
399 13. Drawer S, Fuller CW. Propensity for osteoarthritis and lower limb joint pain in retired professional
400 soccer players. *Br J Sports Med*. 2001. doi:10.1136/bjsm.35.6.402
401 14. Turner AP, Barlow JH, Heathcote-Elliot C. Long term health impact of playing professional football
402 in the United Kingdom. *Br J Sports Med*. 2000. doi:10.1136/bjsm.34.5.332
403 15. Kuijt MTK, Inklaar H, Gouttebarge V, Frings-Dresen MHW. Knee and ankle osteoarthritis in former
404 elite soccer players: A systematic review of the recent literature. *J Sci Med Sport*. 2012.
405 doi:10.1016/j.jsams.2012.02.008
406 16. Davies MAM, Judge DA, Delmestri A, et al. Health amongst former rugby union players: A cross-
407 sectional study of morbidity and health-related quality of life. *Sci Rep*. 2017. doi:10.1038/s41598-
408 017-12130-y
409 17. Schuring N, Aoki H, Gray J, Kerkhoffs GMMJ, Lambert M, Gouttebarge V. Osteoarthritis is
410 associated with symptoms of common mental disorders among former elite athletes. *Knee
411 Surgery, Sport Traumatol Arthrosc*. 2017. doi:10.1007/s00167-016-4255-2
412 18. Gouttebarge V, Inklaar H, Frings-Dresen MHW. Risk and consequences of osteoarthritis after a
413 professional football career: A systematic review of the recent literature. *J Sports Med Phys
414 Fitness*. 2014.
415 19. Vandenbroucke JP, von Elm E, Altman DG, et al. Strengthening the Reporting of Observational
416 Studies in Epidemiology (STROBE): Explanation and elaboration. *Int J Surg*. 2014.
417 doi:10.1016/j.ijsu.2014.07.014

For peer review only - http://bmjopen.bmj.com/site/about/guidelines.xhtml

20. Woodward M. *Epidemiology: Study Design and Data Analysis, Third Edition*.; 2013.

21. National Institute for Health and Care Excellene. National Clinical Guideline Centre Osteoarthritis Care and management in adults. 2014;(February):1-505.

22. Hays RD, Bjorner JB, Revicki DA, Spritzer KL, Cella D. Development of physical and mental health summary scores from the patient-reported outcomes measurement information system (PROMIS) global items. *Qual Life Res*. 2009. doi:10.1007/s11136-009-9496-9

23. Felson DT. An update on the pathogenesis and epidemiology of osteoarthritis. *Radiol Clin North Am*. 2004. doi:10.1016/S0033-8389(03)00161-1

24. DJ H, JJ M, FJ K. The symptoms of osteoarthritis and the genesis of pain. *Med Clin North Am*. 2009. doi:10.1016/j.mcna.2008.08.008

25. Gouttebarge V, Castaldelli-Maia JM, Gorczynski P, et al. Occurrence of mental health symptoms and disorders in current and former elite athletes: A systematic review and meta-analysis. *Br J Sports Med*. 2019. doi:10.1136/bjsports-2019-100671

26. Gross P, Marti B. Risk of degenerative ankle joint disease in volleyball players: Study of former elite athletes. *Int J Sports Med*. 1999. doi:10.1055/s-2007-971094

27. Konradsen L, Berg Hansen EM, Søndergaard L. Long distance running and osteoarthrosis. *Am J Sports Med*. 1990. doi:10.1177/036354659001800408

28. Schmitt H, Lemke JM, Brocai DRC, Parsch D. Degenerative changes in the ankle in former elite high jumpers. *Clin J Sport Med*. 2003. doi:10.1097/00042752-200301000-00002

29. Niek Van Dijk C, Lim LSL, Poortman A, Strübbe EH, Marti RK. Degenerative Joint Disease in Female Ballet Dancers. *Am J Sports Med*. 1995. doi:10.1177/036354659502300307

30. Murray Leslie CF, Lintott DJ, Wright V. The knees and ankles in sport and veteran military parachutists. *Ann Rheum Dis*. 1977. doi:10.1136/ard.36.4.327

31. Menz HB, Munteanu SE, Landorf KB, Zammit G V., Cicuttini FM. Radiographic classification of

For peer review only

For peer review only - http://bmjopen.bmj.com/site/about/guidelines.xhtml

osteoarthritis in commonly affected joints of the foot. *Osteoarthr Cartil*. 2007. doi:10.1016/j.joca.2007.05.007

32. Wikstrom EA, Hubbard-Turner T, McKeon PO. Understanding and Treating Lateral Ankle Sprains and their Consequences: A Constraints-Based Approach. *Sport Med*. 2013. doi:10.1007/s40279-013-0043-z

33. Filbay S, Pandya T, Thomas B, McKay C, Adams J, Arden N. Quality of Life and Life Satisfaction in Former Athletes: A Systematic Review and Meta-Analysis. *Sport Med*. 2019;(0123456789). doi:10.1007/s40279-019-01163-0

34. Arnold R, Fletcher D. A Research Synthesis and Taxonomic Classification of the Organizational Stressors Encountered by Sport Performers. *J Sport Exerc Psychol*. 2016. doi:10.1123/jsep.34.3.397

35. Wylleman P, Reints A. A lifespan perspective on the career of talented and elite athletes: Perspectives on high-intensity sports. *Scand J Med Sci Sport*. 2010. doi:10.1111/j.1600-0838.2010.01194.x

36. Nouni-Garcia R, Carratala-Munuera C, Orozco-Beltran D, Lopez-Pineda A, Asensio-Garcia MR, Gil-Guillen VF. Clinical benefit of the FIFA 11 programme for the prevention of hamstring and lateral ankle ligament injuries among amateur soccer players. *Inj Prev*. 2018. doi:10.1136/injuryprev-2016-042267

37. Attwood MJ, Roberts SP, Trewartha G, England ME, Stokes KA. Efficacy of a movement control injury prevention programme in adult men’s community rugby union: a cluster randomised controlled trial. *Br J Sports Med*. 2018. doi:10.1136/bjsports-2017-098005

38. Kirkendall DT, Garrett WE. Management of the Retired Athlete with Osteoarthritis of the Knee. *Cartilage*. 2012. doi:10.1177/1947603511408287

39. McAlindon TE, Bannuru RR, Sullivan MC, et al. OARSI guidelines for the non-surgical management

For peer review only

For peer review only - http://bmjopen.bmj.com/site/about/guidelines.xhtml

of knee osteoarthritis. *Osteoarthr Cartil*. 2014. doi:10.1016/j.joca.2014.01.003

40. Paterson KL, Gates L. Clinical Assessment and Management of Foot and Ankle Osteoarthritis: A Review of Current Evidence and Focus on Pharmacological Treatment. *Drugs and Aging*. 2019. doi:10.1007/s40266-019-00639-y

41. Gouttebarge V, Goedhart E, Kerkhoffs G. Empowering the health of retired professional footballers: The systematic development of an after Career Consultation and its feasibility. *BMJ Open Sport Exerc Med*. 2018. doi:10.1136/bmjsem-2018-000466

For peer review only

STROBE Statement—Checklist of items that should be included in reports of ***cross-sectional studies***

	Item No	Recommendation
**Title and abstract**	1	(*a*) Indicate the study's design with a commonly used term in the title or the abstract – √
		(*b*) Provide in the abstract an informative and balanced summary of what was done and what was found – √
**Introduction**		
Background/rationale	2	Explain the scientific background and rationale for the investigation being reported – √: p. 4 line 64 – 96
Objectives	3	State specific objectives, including any prespecified hypotheses – √: p. 4 line 98 – 104
**Methods**		
Study design	4	Present key elements of study design early in the paper – √: p. 6 line 108 – 114
Setting	5	Describe the setting, locations, and relevant dates, including periods of recruitment, exposure, follow-up, and data collection – √: p. 6 line 116 – 133
Participants	6	(*a*) Give the eligibility criteria, and the sources and methods of selection of participants – √: p. 6 line 116 – 133
Variables	7	Clearly define all outcomes, exposures, predictors, potential confounders, and effect modifiers. Give diagnostic criteria, if applicable – √: p. 6 line 135 – 159
Data sources/ measurement	8*	For each variable of interest, give sources of data and details of methods of assessment (measurement). Describe comparability of assessment methods if there is more than one group – √: p. 6 line 161 – 172
Bias	9	Describe any efforts to address potential sources of bias – √: p. 6 line 117
Study size	10	Explain how the study size was arrived at– √: p. 6 – 7 line 130 – 133
Quantitative variables	11	Explain how quantitative variables were handled in the analyses. If applicable, describe which groupings were chosen and why – √: p. 8-9 line 175 – 188
Statistical methods	12	(*a*) Describe all statistical methods, including those used to control for confounding
		(*b*) Describe any methods used to examine subgroups and interactions
		(*c*) Explain how missing data were addressed
		(*d*) If applicable, describe analytical methods taking account of sampling strategy
		(*e*) Describe any sensitivity analyses **– √: p. 8-9 line 175 – 188**
**Results**		
Participants	13*	(a) Report numbers of individuals at each stage of study—eg numbers potentially eligible, examined for eligibility, confirmed eligible, included in the study, completing follow-up, and analysed **– √: p. 10 line 192 – 196**
		(b) Give reasons for non-participation at each stage **– √: p. 10 line 192 – 196**
		(c) Consider use of a flow diagram
Descriptive data	14*	(a) Give characteristics of study participants (eg demographic, clinical, social) and information on exposures and potential confounders **– √: p 11 table 1**
		(b) Indicate number of participants with missing data for each variable of interest
Outcome data	15*	Report numbers of outcome events or summary measures **– √: p 11 – 14, table 2 -4**
Main results	16	(*a*) Give unadjusted estimates and, if applicable, confounder-adjusted estimates and their precision (eg, 95% confidence interval). Make clear which confounders were adjusted for and why they were included **– √: p 11 – 14, table 2 -4**
		(*b*) Report category boundaries when continuous variables were categorized

For peer review only

For peer review only - http://bmjopen.bmj.com/site/about/guidelines.xhtml

		(*c*) If relevant, consider translating estimates of relative risk into absolute risk for a meaningful time period
Other analyses	17	Report other analyses done—eg analyses of subgroups and interactions, and sensitivity analyses **– √: p 15, line 227 – 237 + p 16 table 5**
**Discussion**		
Key results	18	Summarise key results with reference to study objectives **– √: p 17, line 242 - 245**
Limitations	19	Discuss limitations of the study, taking into account sources of potential bias or imprecision. Discuss both direction and magnitude of any potential bias **– √: p 3, line 53 - 62**
Interpretation	20	Give a cautious overall interpretation of results considering objectives, limitations, multiplicity of analyses, results from similar studies, and other relevant evidence **– √: p 19 - 20, line 293 - 317**
Generalisability	21	Discuss the generalisability (external validity) of the study results **p 19 - 20, line 293 - 305**
**Other information**		
Funding	22	Give the source of funding and the role of the funders for the present study and, if applicable, for the original study on which the present article is based **p 21, line 339 - 340**

*Give information separately for exposed and unexposed groups.

Note: An Explanation and Elaboration article discusses each checklist item and gives methodological background and published examples of transparent reporting. The STROBE checklist is best used in conjunction with this article (freely available on the Web sites of PLoS Medicine at http://www.plosmedicine.org/, Annals of Internal Medicine at http://www.annals.org/, and Epidemiology at http://www.epidem.com/). Information on the STROBE Initiative is available at www.strobe-statement.org.

For peer review only

BMJ Open

Ankle osteoarthritis and its association with severe ankle injuries, ankle surgeries and health-related quality of life in recently retired professional male football and rugby players: A Cross-sectional observational study

Journal:	*BMJ Open*
Manuscript ID	bmjopen-2020-036775.R1
Article Type:	Original research
Date Submitted by the Author:	26-Mar-2020
Complete List of Authors:	Paget, Liam; Amsterdam UMC - Locatie AMC, Orthopaedic surgery; Amsterdam Collaboration for Health and Safety in Sports, AMC/VUMC IOC Research Center (ACHSS)<br>Aoki, Haruhito; St. Marianna University School of Medicine; Yokohama City Sports Medical Center<br>Kemp, Simon; Rugby Football Union, Sports Medicine<br>Lambert, Mike; University of Cape Town, UCT/MRC Research Unit for Exercise Science and Sports Medicine<br>Readhead, Clint; South Africa Rugby Union, Medical; University of Cape Town Division of Exercise Science and Sports Medicine<br>Stokes, Keith; University of Bath, Department for Health; Rugby Football Union,<br>Viljoen, Wayne; South African Rugby Union, Medical; University of Cape Town Division of Exercise Science and Sports Medicine<br>Reurink, G; Sports Medicine, OLVG, The Sport Physician Group; Academic Center for Evidence-based Sports medicine (ACES)<br>Tol, J; Academic Center for Evidence-Based Sports medicine (ACES); Aspetar Orthopaedic and Sports Medicine Hospital<br>Kerkhoffs, Gino; Amsterdam UMC - Locatie AMC, Orthopaedic surgery; Amsterdam Collaboration for Health and Safety in Sports (ACHSS)<br>Gouttebarge, Vincent; Amsterdam UMC - Locatie AMC, Orthopaedic surgery; Football Players Worldwide (FIFPRO)
**Primary Subject Heading**:	Sports and exercise medicine
Secondary Subject Heading:	Rehabilitation medicine, Sports and exercise medicine
Keywords:	EPIDEMIOLOGY, ORTHOPAEDIC & TRAUMA SURGERY, Foot & ankle < ORTHOPAEDIC & TRAUMA SURGERY, Orthopaedic sports trauma < ORTHOPAEDIC & TRAUMA SURGERY, SPORTS MEDICINE

SCHOLARONE™
Manuscripts

I, the Submitting Author has the right to grant and does grant on behalf of all authors of the Work (as defined in the below author licence), an exclusive licence and/or a non-exclusive licence for contributions from authors who are: i) UK Crown employees; ii) where BMJ has agreed a CC-BY licence shall apply, and/or iii) in accordance with the terms applicable for US Federal Government officers or employees acting as part of their official duties; on a worldwide, perpetual, irrevocable, royalty-free basis to BMJ Publishing Group Ltd ("BMJ") its licensees and where the relevant Journal is co-owned by BMJ to the co-owners of the Journal, to publish the Work in this journal and any other BMJ products and to exploit all rights, as set out in our licence.

The Submitting Author accepts and understands that any supply made under these terms is made by BMJ to the Submitting Author unless you are acting as an employee on behalf of your employer or a postgraduate student of an affiliated institution which is paying any applicable article publishing charge ("APC") for Open Access articles. Where the Submitting Author wishes to make the Work available on an Open Access basis (and intends to pay the relevant APC), the terms of reuse of such Open Access shall be governed by a Creative Commons licence – details of these licences and which Creative Commons licence will apply to this Work are set out in our licence referred to above.

Other than as permitted in any relevant BMJ Author's Self Archiving Policies, I confirm this Work has not been accepted for publication elsewhere, is not being considered for publication elsewhere and does not duplicate material already published. I confirm all authors consent to publication of this Work and authorise the granting of this licence.

For peer review only

Ankle osteoarthritis and its association with severe ankle injuries, ankle surgeries and health-related quality of life in recently retired professional male football and rugby players: A Cross-sectional observational study

Liam D. A. Paget[1,2,3], Haruhito Aoki[4, 5], Simon Kemp[6], Mike Lambert[7], Clint Readhead[7, 8], Keith Stokes[6, 9, 10], Wayne Viljoen[7, 8], Gustaaf Reurink[1,2,3,11], Johannes L. Tol[2,3,12], Gino M. M. J. Kerkhoffs[1,2,3], Vincent Gouttebarge[1,2,3,4,13]

[1] Amsterdam UMC, University of Amsterdam, Department of Orthopaedic Surgery, Amsterdam Movement Sciences, Meibergdreef 9, Amsterdam, the Netherlands

[2] Academic Center for Evidence based Sports medicine (ACES), The Netherlands; Amsterdam

[3] Amsterdam Collaboration on Health & Safety in Sports (ACHSS), AMC/VUmc IOC Research Center of Excellence, Amsterdam, The Netherlands

[4] St. Marianna University School of Medicine, Kawasaki, Japan

[5] Yokohama City Sports Medical Center, Yokohama, Japan

[6] Rugby Football Union, Twickenham, UK

[7] Division of Exercise Science and Sports Medicine (ESSM), University of Cape Town, Cape Town, South Africa

[8] South African Rugby Union (SARU), Cape Town, South Africa

[9] Department for Health, University of Bath, Bath, UK

[10] Centre for Sport, Exercise and Osteoarthritis Research Versus Arthritis

[11] The Sport Physician Group, Department of Sports Medicine, OLVG, Amsterdam, The Netherlands

[12] Aspetar, Orthopaedic and Sports Medicine Hospital, Doha, Qatar

[13] Football Players Worldwide (FIFPRO), Hoofddorp, The Netherlands

Correspondence

Liam. D.A. Paget – L.d.paget@amsterdamumc.nl

Department of Orthopaedic Surgery, Amsterdam UMC

PO Box 22660, 1100 DD Amsterdam, The Netherlands

For peer review only

Abstract

Objectives

To determine (i) the prevalence of ankle osteoarthritis (OA) among former professional football and rugby players, (ii) assess the association between ankle injuries or ankle surgeries with ankle OA, and (iii) compare the mental and physical quality of life (QoL) between former professional football and rugby players with and without OA.

Methods

We conducted a questionnaire based observational study with a cross-sectional design. Former professional football and rugby players were recruited by the football players worldwide (FIFPRO) and the International Rugby Players (IRP). Information concerning ankle OA, sustained ankle injuries and ankle surgeries was gathered (medical record or most recent medical professional). Health related QoL was assessed using the PROMIS physical and mental health scores.

Results

Overall, 553 former professional football (n = 401) and rugby players (n = 152) were enrolled in the study (response rate of 56%). Ankle OA prevalence among former professional football and rugby players was 9.2% and 4.6%, respectively. Football players were more likely to suffer from ankle OA following every ankle injury and/or surgery. Football and rugby players with ankle OA had similar PROMIS physical and mental health scores to the norm for the general population.

Conclusion

Former professional football and rugby players had a higher ankle OA prevalence than the general population (3.4%). Football players are more likely to suffer from ankle OA following every ankle injury

For peer review only
For peer review only - http://bmjopen.bmj.com/site/about/guidelines.xhtml

and/or surgery. No clinical relevant difference was seen for physical or mental health related QoL among football and rugby players. Preventive measures for ankle injuries are recommended.

Strengths and limitations

- Overall good response rate (56%) and a large group of participating football players (401)
- This study had a cross-sectional design, therefore no causal-association can be determined
- This study contributes to the literature, where the current available data concerning ankle OA in professional football and rugby players is outdated or non-existent.
- The data is based on (retrospective) questionnaires and is therefore dependent on the accuracy and understanding of the participant. Our OA definition did not include a clinical and radiological evaluation.

For peer review only - http://bmjopen.bmj.com/site/about/guidelines.xhtml

Background

Football and Rugby Union (hereafter 'rugby') are among the most popular team sports worldwide. In both sports, ankle injuries represent a significant proportion of the total injuries sustained.[1–5] Previous ankle injuries are an important risk factor for ankle osteoarthritis (OA).[6,7]

Drawer et al. (2002) estimates the overall risk of injury in professional football to be 1,000 times higher than in high-risk industrial occupations, with ankle injuries contributing 10-18% of all injuries.[2,8] The Union of European Football Associations (UEFA)Champions League Injury Study reported an overall ankle injury rate of 1 injury per 1000 hours.[2] Lateral ankle sprains (51%) were the most prevalent sub-type of ankle injuries.[2] In professional rugby, epidemiological studies show that ankle injuries contribute 5-20% of all injuries.[1,3,9,10] Similar to professional football, lateral ankle sprains are the most common ankle injuries with an injury rate of 3.8 - 4.5 injuries per 1,000 player hours depending on the position on the field.[11]

Two retrospective cohort studies from the general population (n = 390 and n = 639 patients respectively), reported that 70-78% of ankle OA cases were associated with a previously sustained ankle injury. These previous injuries included fractures, isolated osteochondral defects of the talus and ankle ligament injuries.[6,7] Among these post-traumatic ankle OA cases, 28% were associated with a previously sustained ligamentous injury.[7] To our knowledge, there are no prospective studies on the association of ankle injuries and ankle OA.

In the general population the prevalence of ankle OA is estimated to be 3.4% in people over 50 years of age. [12] The reported prevalence of ankle OA 16 to 22 years after a professional football career is 12-19%.[13–15] No data are available for ankle OA among former professional rugby players. In comparison to

For peer review only - http://bmjopen.bmj.com/site/about/guidelines.xhtml

a general population control group, the prevalence of OA in any joint and the incidence of joint replacement for any joint was estimated to be 4 and 6 times higher, respectively, among former elite rugby players.[16] This has been associated with a significantly poorer mental and physical health-related quality of life (QoL).[16]

Several other studies report the association of general OA with mental health symptoms and physical health-related QoL among former elite athletes across professional team sports including football and rugby.[17,18] Therefore, the impact of OA on retired players must be considered. To date, the epidemiological evidence concerning the prevalence of ankle OA in professional football is either outdated or limited, and in the case of rugby non-existent, while data on the association between previous ankle injury and ankle OA is scarce in both sports.

The objective of this study was threefold. Firstly, to evaluate the prevalence of ankle OA in recently retired professional football and rugby players. Secondly, to determine the association between ankle injury and/or ankle surgery and ankle OA. Thirdly, to compare the mental and physical QoL in players with and without ankle OA. Our hypotheses were that (i) recently retired professional football and rugby players have a higher ankle OA prevalence than the general population (as found in the literature), (ii) ankle injuries and/or ankle surgeries are associated with ankle OA, and (iii) the mental and physical QoL of recently retired professional football and rugby players with OA is lower compared to those players without OA.

For peer review only - http://bmjopen.bmj.com/site/about/guidelines.xhtml

Methods

Design

We conducted a questionnaire based observational study with a cross-sectional design. Ethical approval for the study was provided by the Ethical Committee of the Yokohama City Sports Medical Center (17.003; Yokohama, Japan) and the Medical Ethics Review Committee of the Academic Medical Center (W16_366#16.431; Amsterdam, The Netherlands). The study was reported according to the 'Strengthening the Reporting of Observational Studies in Epidemiology' statement and conducted in accordance with the Declaration of Helsinki (2013).[19]

Participants

Retired professional football and rugby players were randomly recruited to reduce recruitment bias, by the Football Players Worldwide (FIFPRO) and the International Rugby Players (IRP). Inclusion criteria were: (a) being a retired male professional football or rugby player; (b) being younger than 50 years of age; (c) being able to read and comprehend texts in English, French or Spanish.

The definition of a retired professional football or rugby player was that he (i) has trained to improve performances, (ii) has competed in the highest or second highest national league, and (iii), has had training and competition as a major activity (way of living) or focus of personal interest, devoting several hours in all or most of the days for these activities, and exceeding the time allocated to other types of professional or leisure activities. If interested, players received information about the study, gave their informed consent and completed an electronic questionnaire. Once completed, the electronic questionnaires were saved automatically on a secured electronic server that only the principal investigator could access.

Sample size calculation for ankle OA prevalence indicated that 138 participants per sport were needed

For peer review only - http://bmjopen.bmj.com/site/about/guidelines.xhtml

For peer review only

134 (power of 80%, confidence interval of 95%; absolute precision of 5%) under the assumption of an anticipated population proportion of 10%.[20] Expecting a response rate of approximately 50%, we intended to reach at least 300 participants per sport.

Dependent variable: Ankle osteoarthritis

The clinical diagnosis of ankle OA by a medical professional was retrospectively determined using a single question ('Have you been diagnosed with ankle osteoarthritis by a medical professional?'). Participants were given the definition of ankle OA (based on the NICE criteria; adapted for age), determined as the damage of the ankle joint's cartilage that leads to activity-related joint pain with either no morning joint-related stiffness or morning stiffness that lasts no longer than 30 minutes.[21] For this question, participants were requested to consult either their medical record or their most recent medical professional.

Independent variable: Severe ankle injury and related surgery

History of a severe ankle injury during their professional football or rugby career was examined through a single question for the sequential recording of single or multiple injury events over time. Similarly, history of an ankle related surgery was recorded through a single question for the sequential recording of a single or multiple ankle surgeries over time. In our study, a severe ankle injury was defined as an injury that involved the ankle joint, occurred during team activities (training or match), and led to absence from either training or a match for more than 28 days [18,19]. For this question, participants were requested to consult either their medical records or their most recent medical professional.

Health-related quality of life

The Patient-Reported Outcomes Measurement Information System Global Health short form (PROMIS-GH) assesses multiple domains related to health-related quality of life such as health, functioning, pain,

For peer review only - http://bmjopen.bmj.com/site/about/guidelines.xhtml

social activities and fatigue.[20] The PROMIS-GH has been validated in several populations and languages among which are English, French and Spanish (for detailed information, see www.nihpromis.org).[20] The Global Mental Health and Global Physical Health scores were calculated based on 10 items each measured on a 5-point scale (from 1 to 5) and subsequently converted.[20] These subscale scores ranged from 0 to 100, with a higher score indicating better quality of life and a mean score of 50 indicating the norm for the general population.[22]

Procedures

An anonymous questionnaire (electronic and/or paper) available in English, French and Spanish was compiled (LimeSurvey Professional). It included the following descriptive variables: age, body-height, body-weight, duration of professional football or rugby career, level of play, duration and nature of retirement, and current employment status (employed or not employed). Additionally, this questionnaire included questions for the outcomes previously elaborated on (ankle OA, ankle injury, ankle surgery, QoL). Information about the study was sent per email to potential participants by FIFPRO and IRP. Participants interested in the study, gave their informed consent and completed the electronic questionnaire. Participants were asked to complete the questionnaire within 2 weeks, reminders being sent after 2 and 4 weeks. For privacy reasons, the responses to the questionnaires were coded and anonymized. Once completed, the electronic questionnaires were automatically saved on a secured electronic server that only the principal investigator could access. Players participated voluntarily and did not receive any reward for their participation.

Statistical analyses

Data analysis was performed using the statistical software IBM SPSS 24.0 for Windows. Analyses were conducted separately for retired professional football and rugby players. Data were assessed (visually) for

For peer review only - http://bmjopen.bmj.com/site/about/guidelines.xhtml

For peer review only

182 normality and presented as mean ± standard deviation (SD), median (interquartile range [IQR]), or frequency (proportion in %) as appropriate. Body mass index (BMI) was calculated using the provided weight and height of participants (kg/m²). Prevalence of ankle OA, overall and within the age categories of ≤40 years and >40 years, was calculated as the proportion of the number of participants with ankle OA relative to the total number of participants in each category.[20] *We chose to dichotomize age at 40 years, as our population included former professional athletes. This is opposed to Murray et al. (2018) concerning the general population (dichotomized at 50 years of age), and in line with Song et al. (2019) studying former professional American Football players. We made the assumption that although no-longer professionals, these former professional athletes would no longer be competing at a decent level at the age of 40. We therefore found 40 years to be a more relevant age to dichotomise than 50 years.*[12,23] A logistic regression analysis was performed to determine the association of the number of severe ankle injuries and/or ankle surgeries (continuous independent variable) with ankle OA (dichotomous dependent variable). Severe ankle injuries and or ankle-related surgeries were also expressed using 3 pre-determined categories (0, 1 and >1). This was adjusted for age and BMI, both having been identified as risk factors for OA.[24,25] Descriptive analyses of health-related quality of life (Global Physical Health and Global Mental Health) were conducted, while comparisons between groups (retired players with ankle OA vs. retired players without ankle OA) were made using a Mann-Whitney U test for independent samples.[20]

Patient and public involvement

This research was done without patient involvement. Patients were not invited to comment on the study design and were not consulted to develop patient relevant outcomes or interpret the results. Patients were not invited to contribute to the writing or editing of this document for readability or accuracy.

For peer review only

Results

Participants

From a total of 750 football and 326 rugby players contacted, respectively 401 and 152 gave their written informed consent and completed the questionnaire (overall response rate of 56%). Characteristics of the two groups including the pre-determined sub-categories for severe ankle injuries and ankle surgeries (0, 1 and >1) are illustrated in Table 1.

Prevalence of ankle osteoarthritis, severe injury and surgery

The prevalence of patient reported ankle OA (including the pre-determined categories of ≤ 40 years and > 40 years) among recently retired professional football and rugby players, is presented in Table 2. The prevalence of ankle OA in former professional football players was 9.2% (n = 37). Overall, 94% of players with ankle OA were documented to have sustained ≥1 ankle injury and 62% had undergone ≥1 ankle surgery. In the non-OA group (n = 364), 54% had sustained ≥1 ankle injury and 14% had undergone ≥1 ankle surgery. Logistic regression analysis (Table 3) showed that football players who had sustained a severe ankle injury were 1.3 (95%CI: 1.1 - 1.6) times more likely to report ankle OA ($p < 0.001$). Football players who had undergone ankle surgery were 2.1 (95%CI: 1.4 – 3.1) times more likely to report ankle OA ($p < 0.001$).

The prevalence of ankle OA in former professional rugby players was 4.6% (n = 7). Overall, six out of seven (86%) rugby players with ankle OA reported to have sustained ≥1 ankle injury and four out of seven (57.1%) had undergone ≥1 ankle surgery. In the non-OA group (n = 145), 58.2% had sustained ≥1 ankle injury and 18.4% had undergone ≥1 ankle surgery. Logistic regression analysis (Table 3) showed no significant association between either severe ankle injuries (OR = 1.1 95%CI: 0.8 – 1.5) or ankle surgeries (OR = 2.0 95%CI: 0.8 – 4.7) and ankle OA among former professional rugby players.

For peer review only - http://bmjopen.bmj.com/site/about/guidelines.xhtml

For peer review only

Table 1: Descriptive characteristics of recently retired professional football players and rugby players

	Football			Rugby		
	Total (n = 401)	Ankle OA (n = 37)	No ankle OA (n = 364)	Total (n = 152)	Ankle OA (n = 7)	No ankle OA (n = 145)
*Age in years * (median; IQR; min-max / mean ± SD)*	36 (32-40; 25-50)	39 (36-42; 27-49)*	36 (32-40; 25-50)*	40 ± 6	41 ± 7	40 ± 6
Height in cm (mean ± SD / median; IQR; min-max)	181 ± 7	180 ± 7	182 ± 7	186 (180-191; 166-203)	191 (173-192; 172-192)	186 (180-191; 166-203)
Weight in kg (in kg (mean ± SD))	82 ± 9	82 ± 13	82 ± 9	100 (91-114; 59-170)	108 (89-123; 82-130)	100 (91-113; 59-170)
BMI in kg/m² (median; IQR; min-max))	24.8 (23.5-26.2; 20.1-32.7)	24.8 (22.9-26.6; 20.3-29)	24.7 (23.5-26.1; 20.1-32.7)	29.0 (27.1-31.4; 20.4-52.5)	32.2 (28.5-33.7; 27.4-35.3)*	28.9 (27.0-31.2; 20.4-52.5)*
Employed in % (n)	89% (350)	92% (33)	89% (317)	91% (139)	86% (6)	92% (133)
Professional career duration in years (median; IQR; min-max)	13 (10-15; 2-19)	13 (12-16; 5-18)	13 (9-15; 2-19)	10 (7-15; 2-20)	10 (8-12.5; 8-13)	10 (7-15; 2-20)
Retirement duration in years (median; IQR; min-max)	4 (2-7; 1-17)	5 (3-9; 1-12)	4 (2-7; 1-17)	8 (3-12; 0-22)	12 (2-16; 1-19)	8 (4-11; 0-22)
Level of play top league; % (n)	81% (314)	92% (34)	80% (280)	80% (106)	86% (6)	80% (100)
Forced retirement % (n)	29% (113)	27% (10)	30% (103)	36% (55)	57% (4)	35% (51)
Severe ankle injuries (median; min-max)	1 (0-2; 0-10)	2 (1-3; 0-10)	1 (0-2; 0-10)	1 (0-2; 0-20)	1 (1-2; 0-3)	1 (0-2; 0-20)
Ankle surgeries (median; IQR; min-max)	0 (0-0; 0-5)	1 (0-2; 0-5)	0 (0-0; 0-5)	0 (0-0; 0-4)	1 (0-1; 0-2)	0 (0-0; 0-4)

For peer review only - http://bmjopen.bmj.com/site/about/guidelines.xhtml

* Statistically significant difference between ankle OA and no ankle OA. Abbreviations: OA = Osteoarthritis; IQR = interquartile range; n = number

For peer review only - http://bmjopen.bmj.com/site/about/guidelines.xhtml

BMJ Open

Table 2: Subcategories for prevalence of reported ankle OA, severe injuries and ankle surgeries in retired football and rugby players

	**Football**			**Rugby**		
	Total (n = 401)*	≤ 40 years (n = 271)	> 40 years (n = 83)	Total (n = 152)	≤ 40 years (n = 82)	> 40 years (n = 66)
Prevalence ankle OA in % (95%CI)	9.2% (6.4 –12.1)	8.5% (5.2 – 11.8)	12.0% (5.0 – 19.1)	4.6% (1.2 – 8.0)	2.4% (0.9 – 5.8)	4.5% (0.5 – 9.6)
	0 ankle injuries	1 ankle injury	> 1 ankle injury	0 ankle injuries	1 ankle injury	> 1 ankle injury
Severe ankle injuries total (%, (n))	42.5% (156)	25.3% (93)	32.2% (118)	40.5% (60)	27.7% (41)	31.8% (47)
- *With ankle OA (%, (n))*	5.7% (2)	28.6% (10)	65.7% (23)	14.3% (1)	42.9% (3)	42.9% (3)
- *Without ankle OA (%, (n))*	46.4% (154)	25.0% (83)	28.6% (95)	41.8% (59)	27.0% (38)	31.2% (44)
	No ankle surgeries	1 ankle surgery	> 1 ankle surgery	No ankle surgeries	1 ankle surgery	> 1 ankle surgery
Ankle surgeries total in % (n)	81.5% (299)	10.9% (40)	7.6% (28)	79.7% (118)	15.5% (23)	4.7% (7)
- *With ankle OA % (n)*	38.2% (13)	29.4% (10)	32.4% (11)	42.9% (3)	42.9% (3)	14.3% (1)
- *Without ankle OA % (n)*	85.9% (286)	9% (30)	5.1% (17)	81.6% (115)	14.2% (20)	4.3% (6)

**The sum of patients in the subcategories ≤ 40 years and > 40 years does not equal the "Total number of participants" due to missing data (regarding age).*
Abbreviations: OA = Osteoarthritis; n = number; CI = confidence interval;

For peer review only - http://bmjopen.bmj.com/site/about/guidelines.xhtml

Table 3: Association (odds ratio and 95% CI) of severe ankle injury and ankle surgery with ankle osteoarthritis among recently retired professional football and rugby players

	Football (n = 401)		Rugby (n = 152)	
	Unadjusted	Adjusted$^{\psi}$	Unadjusted	Adjusted$^{\psi}$
Severe ankle injury (OR, 95%CI)	1.34 (1.17-1.53)	1.33 (1.14-1.55)	1.01 (0.74-1.38)	1.05 (0.75-1.47)
Ankle surgery (OR, 95%CI)	2.20 (1.61-3.00)	2.09 (1.41-3.08)	1.99 (0.92-4.3)	1.96 (0.81-4.71)

$^{\psi}$Adjusted for age and body mass index (BMI); Abbreviations: OR = Odds Ratio; n = number; CI = confidence interval

For peer review only

For peer review only - http://bmjopen.bmj.com/site/about/guidelines.xhtml

Mental and physical QoL

For the PROMIS physical health score a statistically significant lower outcome was found for football (p = 0.01) and rugby players (p = 0.009) with OA (48 and 45 points respectively) compared to players without OA (51 and 51 points respectively). The PROMIS mental health score was not significantly different between players with OA vs players without OA. Data is presented in Table 4.

Table 4: Mental and physical quality of life (0-100) of recently retired football and rugby players with ankle OA compared to players without ankle OA

	**Football**				**Rugby**			
	Total (n = 401)	With ankle OA (n = 37)	Without ankle OA (n = 364)	p-value	Total (n = 152)	With ankle OA (n = 7)	Without ankle OA (n = 145)	p-value
PROMIS mental health (median; IQR; min-max)	51; 46-56; 31-68	51; 46-53; 31-63	53; 46-56; 31-68	0.251	52; 46-56; 28-68	48; 48-53; 46-56	53; 46-56; 28-68	0.567
PROMIS Physical health (median; IQR; min-max)*	51; 46-56; 27-68	48; 42-54; 35-62*	51; 48-58; 27-68*	0.010	51; 45-54; 30-68	45; 37-48; 37-51*	51; 37-56; 30-68*	0.009

**Statistically significant difference;* Abbreviations: OA = Osteoarthritis; IQR = Interquartile range; n = number

For peer review only - http://bmjopen.bmj.com/site/about/guidelines.xhtml

Post-hoc analysis

In the literature, forced retirement is a risk factor for post-career mental health symptoms.[26] Following data analysis, we were concerned by the seemingly high percentage of football and rugby players who stopped due to a career ending injury. As we were analysing the impact ankle OA had on QoL, we felt the effect of a career ending injury on QoL to be in line with our initial objectives. Consequently we performed a post-hoc analysis to determine any associations between reported forced retirement and mental and physical PROMIS health scores. In this study, 31% of players reported to have been forced to retire, specifically: 29% of football players and 36% of rugby players.

The post-hoc sub-analysis of all players regarding mental and physical health scores associated with voluntary retirement and in conjunction with ankle OA is presented in Table 5. No post-hoc sub-analysis was done for football and rugby players separately, regarding voluntary retirement in conjunction with ankle OA, due to the low number of ankle OA cases among rugby players. However mental and physical health scores in football and rugby due to forced retirement is presented separately in Table 5.

For peer review only - http://bmjopen.bmj.com/site/about/guidelines.xhtml

Table 5: Post-Hoc analysis regarding mental and physical quality of life of recently retired football and rugby players who reported to have been forced to retire, and quality of life of players who reported to have been forced to retire with ankle OA compared to players without ankle OA

	**PROMIS mental health** (median; IQR; min-max)	**p-value**	**PROMIS physical health** (median; IQR; min-max)	**p-value**
Total				
Voluntarily retired (n = 369)*	53; 48-56; 31-68*	p < 0.001	54; 48-58; 30-68*	p < 0.001
Forced to retire (n = 168)*	48; 44-53; 28-68*		48; 42-51; 27-68*	
Voluntarily retired with OA (n = 30)*	51; 46-53; 36-63	p = 0.074	51; 45-54; 35-62*	p = 0.001
Voluntarily retired without OA (n = 339)*	53; 48-59; 31-68		54; 48-58; 30-68*	
Forced to retire with OA (n = 14)	48; 46-55; 31-63	p = 0.841	42; 40-46; 37-58	p = 0.159
Forced to retire without OA (n = 154)	48; 44-53; 28-68		48; 42-52; 27-68	
*Football**				
Voluntarily retired (n = 272)*	53; 48-56; 31-68*	p < 0.001	54; 48-58; 30-68*	p < 0.001
Forced to retire (n = 113)*	48; 44-53; 31-63*		48; 42-51; 27-62*	
*Rugby**				
Voluntarily retired (n = 97)*	53; 48-56; 31-68*	p = 0.018	54; 48-58; 35-68*	p < 0.001
Forced to retire (n = 55)*	48; 44-56; 28-68*		48; 37-54; 30-68*	

**Statistically significant difference; Abbreviations: IQR = Interquartile range; OA = Osteoarthritis; n = number*

For peer review only - http://bmjopen.bmj.com/site/about/guidelines.xhtml

Discussion

Our most important findings were that (i) the prevalence of ankle OA among former professional football and rugby players was, 9.2% (95%CI 6.4 –12.1) and 4.6% (95%Cl (1.2 – 8.0), respectively; (ii) previous severe ankle injuries and ankle surgeries are associated with ankle OA among former professional football players; and (iii) ankle OA does not lead to reduced mental or physical QoL in former professional football and rugby players.

Ankle OA in football and rugby compared to other sports and the general population

Several studies have reported symptomatic ankle OA prevalence among a variety of former elite athletes, ranging from 0% in volleyball (n = 22), long distance running (n = 30), elite high-jump (n = 30) and ballet (n = 27) to 10% among former military parachutists (n = 40).[27–31] In our study, the prevalence of ankle OA among former professional football (9.2%) and rugby players (4.6%) was higher than the estimated prevalence among the general population (3.4 %).[12] Murray et al. (2018) invited participants with self-reported ankle pain (11.7%) from the general population for a radiograph (radiological atlas of Foot OA).[12,32]

Our study reports a lower ankle OA prevalence compared to previous studies among former professional football players (12-19%).[13–15] In these studies, data was similarly collected using cross-sectional retrospective self-reported questionnaires.[13,14] No previous data is available concerning ankle OA among former professional rugby players. The low ankle OA prevalence among rugby players in this study is likely due to either an under reporting of ankle OA among rugby players or a type II error.

Association of severe ankle injuries and ankle surgeries with ankle OA

In our study, football players were 1.3 (95%CI: 1.1 - 1.6) and 2.1 (95%CI: 1.4 – 3.1) times more likely to develop ankle OA for every ankle injury and ankle surgery, respectively. This is in line with previous

For peer review only - http://bmjopen.bmj.com/site/about/guidelines.xhtml

studies that showed an association between ankle OA and ankle injuries, stating traumatic ankle injuries to be the main cause of ankle OA (70-78%).[6,7] Consequences of ankle injuries are thought to be due to either direct damage the articular surfaces or change in ankle joint biomechanics (e.g. through chronic ankle instability due to ankle ligament damage).[33] No association was seen for severe ankle injuries or ankle surgeries and ankle OA among rugby players. Although the best estimate for the association of ankle surgeries and OA is similar to football (OR = 2), the 95%CI includes 1 (0.8 – 4.7). This may be due to insufficient power among rugby players, as only 7 reported ankle OA.

Health-related quality of life

Filbay et al. (2019) reported factors such as age, type of sport (contact/collision sports), OA and involuntary retirement from sport to possibly negatively impact QoL.[26,34–36] However the positive impacts associated with elite sport participation, such as pride of accomplishments, social network (through sport participation) and coping and adjustment to musculoskeletal pain (due to resilience and determination), may persist and even compensate reduced physical QOL beyond retirement of the player's athletic career.[34]

Our post-hoc sub-analysis revealed 29% of football players and 36% of rugby players were forced to retire. Although we reported several instances of statistically significant differences in our study for both physical and mental PROMIS health scores, all were similar to the estimated norm for the general population (50 points). Unfortunately, there is no robust minimally clinically important difference (MCID) threshold for the PROMIS-10 global short form applicable for this population. Consequently, it is difficult to interpret the results. Nevertheless, although statistically significant, the PROMIS mental and physical health scores observed, for both football and rugby players, are unlikely clinically significant.

For peer review only

Based on our data, ankle OA does not lead to reduced mental or physical QoL in former professional football and rugby players.

Strengths and limitations

We had an overall good response rate (56%) and a large group of participating football players (401). However, several limitations should be mentioned. Firstly, this study had a cross-sectional design, therefore no causal-association can be determined. Secondly, there were only 7 rugby players with OA, implying a potential serious type II error. Thirdly, recruitment procedures were blinded to the research team, a non-response analysis could not be performed. Fourthly, research based (retrospective) questionnaires are dependent on the accuracy and understanding of the participant. Professional athletes might generally remember the number of severe ankle injuries or surgeries resulting in a training or match absence of ≥4 weeks. However, we cannot exclude that participants were not able to precisely recall all their sustained severe ankle injuries and ankle surgeries or that they did not consult their most recent medical professional. Finally, our OA definition did not include a clinical and radiological evaluation.

Implications for practice

The epidemiological data from this study regarding ankle OA prevalence among former professional football and rugby players, and its association with both severe ankle injuries and ankle surgeries, is an essential part of protecting the athlete's health. In both professional football and rugby, a higher prevalence of ankle OA can be expected compared to the general population. Furthermore, As the majority are post-traumatic, preventive measures and adequate rehabilitation are recommended. Awareness should be raised, regarding the increased risk of developing ankle OA following each ankle injury and surgery. Additionally primary and secondary prevention programmes should be implemented.

For peer review only - http://bmjopen.bmj.com/site/about/guidelines.xhtml

Exercise based prevention intervention programs, such as the FIFA11+ in football and the movement control injury prevention programme in rugby, have found reduced ankle injury incidence and burden.[37,38] Finally, management of ankle OA is key for retired players, focusing on healthy lifestyle including physical activity, pain alleviation, function and minimising disability.[39–41] With this intention, FIFPRO has developed an "After Career Consultation".[42] Future studies will evaluate its relevance.

Conclusion

This cross-sectional study found an athlete reported ankle OA prevalence of 9.2% and 4.6% among recently retired professional football and rugby players, respectively. Severe ankle injuries and ankle surgeries were associated with a higher prevalence of ankle OA in former professional football players. No clinically relevant differences in QoL were reported in players with OA compared to players without OA. As a higher prevalence of ankle OA can be expected in both professional football and rugby compared to the general population, and the majority are post-traumatic, primary and secondary preventive measures for ankle injuries and an optimal diagnosis and treatment plan are recommended.

Acknowledgements

The Authors would like to thank all football and ruby players' associations involved. The authors are grateful to all retired professional football and rugby players who participated in the study.

Contributorship statement

Authorship was determined according to the ICMJE authorship recommendations. All authors (LP, HA, SK, ML, CR, KS, WV, GR, JT, GK, VG) were involved in the design of the study, data analysis and data interpretation. VG and GK were responsible for data collection. LP drafted the manuscript, with critical review provided by all authors (LP, HA, SK, ML, CR, KS, WV, GR, JT, GK, VG). All

For peer review only - http://bmjopen.bmj.com/site/about/guidelines.xhtml

authors approved the final version of the manuscript (LP, HA, SK, ML, CR, KS, WV, GR, JT, GK, VG).

Funding

This research received no specific grant from any funding agency in the public, commercial or non-profit sectors.

Competing interests

None declared.

Ethics approval

Ethical approval for the study was provided by the Ethical Committee of the Yokohama City Sports Medical Center (17.003; Yokohama, Japan) and the Medical Ethics Review Committee of the Academic Medical Center (W16_366#16.431; Amsterdam, The Netherlands)

Data sharing statement

No additional data available

For peer review only

References

1. Sankey RA, Brooks JHM, Kemp SPT, Haddad FS. The epidemiology of ankle injuries in professional rugby union players. *Am J Sports Med*. 2008. doi:10.1177/0363546508322889
2. Waldén M, Hägglund M, Ekstrand J. Time-trends and circumstances surrounding ankle injuries in men's professional football: An 11-year follow-up of the UEFA Champions League injury study. *Br J Sports Med*. 2013. doi:10.1136/bjsports-2013-092223
3. Fuller CW, Taylor A, Kemp SPT, Raftery M. Rugby World Cup 2015: World Rugby injury surveillance study. *Br J Sports Med*. 2017;51(1):51-57. doi:10.1136/bjsports-2016-096275
4. Ekstrand J, Krutsch W, Spreco A, et al. Time before return to play for the most common injuries in professional football: a 16-year follow-up of the UEFA Elite Club Injury Study. *Br J Sports Med*. 2019:bjsports-2019-100666. doi:10.1136/bjsports-2019-100666
5. Williams S, Trewartha G, Kemp S, Stokes K. A meta-analysis of injuries in senior men's professional Rugby Union. *Sport Med*. 2013. doi:10.1007/s40279-013-0078-1
6. Valderrabano V, Horisberger M, Russell I, Dougall H, Hintermann B. Etiology of ankle osteoarthritis. *Clin Orthop Relat Res*. 2009. doi:10.1007/s11999-008-0543-6
7. Saltzman CL, Salamon ML, Blanchard GM, et al. Epidemiology of ankle arthritis: report of a consecutive series of 639 patients from a tertiary orthopaedic center. *Iowa Orthop J*. 2005.
8. Drawer S, Fuller CW. Evaluating the level of injury in English professional football using a risk based assessment process. *Br J Sports Med*. 2002. doi:10.1136/bjsm.36.6.446
9. Bathgate A, Best JP, Craig G, Jamieson M. A prospective study of injuries to elite Australian rugby union players. *Br J Sports Med*. 2002. doi:10.1136/bjsm.36.4.265
10. Garraway WM, Lee AJ, Hutton SJ, Russell EBAW, Macleod DAD. Impact of professionalism on injuries in rugby union. *Br J Sports Med*. 2000. doi:10.1136/bjsm.34.5.348
11. Brooks JHM, Fuller CW, Kemp SPT, Reddin DB. Epidemiology of injuries in English professional

For peer review only

For peer review only - http://bmjopen.bmj.com/site/about/guidelines.xhtml

rugby union: Part 1 match injuries. *Br J Sports Med*. 2005. doi:10.1136/bjsm.2005.018135

12. Murray C, Marshall M, Rathod T, Bowen CJ, Menz HB, Roddy E. Population prevalence and distribution of ankle pain and symptomatic radiographic ankle osteoarthritis in community dwelling older adults: A systematic review and cross-sectional study. *PLoS One*. 2018. doi:10.1371/journal.pone.0193662

13. Drawer S, Fuller CW. Propensity for osteoarthritis and lower limb joint pain in retired professional soccer players. *Br J Sports Med*. 2001. doi:10.1136/bjsm.35.6.402

14. Turner AP, Barlow JH, Heathcote-Elliot C. Long term health impact of playing professional football in the United Kingdom. *Br J Sports Med*. 2000. doi:10.1136/bjsm.34.5.332

15. Kuijt MTK, Inklaar H, Gouttebarge V, Frings-Dresen MHW. Knee and ankle osteoarthritis in former elite soccer players: A systematic review of the recent literature. *J Sci Med Sport*. 2012. doi:10.1016/j.jsams.2012.02.008

16. Davies MAM, Judge DA, Delmestri A, et al. Health amongst former rugby union players: A cross-sectional study of morbidity and health-related quality of life. *Sci Rep*. 2017. doi:10.1038/s41598-017-12130-y

17. Schuring N, Aoki H, Gray J, Kerkhoffs GMMJ, Lambert M, Gouttebarge V. Osteoarthritis is associated with symptoms of common mental disorders among former elite athletes. *Knee Surgery, Sport Traumatol Arthrosc*. 2017. doi:10.1007/s00167-016-4255-2

18. Gouttebarge V, Inklaar H, Frings-Dresen MHW. Risk and consequences of osteoarthritis after a professional football career: A systematic review of the recent literature. *J Sports Med Phys Fitness*. 2014.

19. Vandenbroucke JP, von Elm E, Altman DG, et al. Strengthening the Reporting of Observational Studies in Epidemiology (STROBE): Explanation and elaboration. *Int J Surg*. 2014. doi:10.1016/j.ijsu.2014.07.014

For peer review only - http://bmjopen.bmj.com/site/about/guidelines.xhtml

20. Woodward M. *Epidemiology: Study Design and Data Analysis, Third Edition*.; 2013.

21. National Institute for Health and Care Excellene. National Clinical Guideline Centre Osteoarthritis Care and management in adults. 2014;(February):1-505.

22. Hays RD, Bjorner JB, Revicki DA, Spritzer KL, Cella D. Development of physical and mental health summary scores from the patient-reported outcomes measurement information system (PROMIS) global items. *Qual Life Res*. 2009. doi:10.1007/s11136-009-9496-9

23. Song K, Wikstrom EA, Tennant JN, Guskiewicz KM, Marshall SW, Kerr ZY. Osteoarthritis Prevalence in Retired National Football League Players With a History of Ankle Injuries and Surgery. *J Athl Train*. 2019. doi:10.4085/1062-6050-421-18

24. Felson DT. An update on the pathogenesis and epidemiology of osteoarthritis. *Radiol Clin North Am*. 2004. doi:10.1016/S0033-8389(03)00161-1

25. DJ H, JJ M, FJ K. The symptoms of osteoarthritis and the genesis of pain. *Med Clin North Am*. 2009. doi:10.1016/j.mcna.2008.08.008

26. Gouttebarge V, Castaldelli-Maia JM, Gorczynski P, et al. Occurrence of mental health symptoms and disorders in current and former elite athletes: A systematic review and meta-analysis. *Br J Sports Med*. 2019. doi:10.1136/bjsports-2019-100671

27. Gross P, Marti B. Risk of degenerative ankle joint disease in volleyball players: Study of former elite athletes. *Int J Sports Med*. 1999. doi:10.1055/s-2007-971094

28. Konradsen L, Berg Hansen EM, Søndergaard L. Long distance running and osteoarthrosis. *Am J Sports Med*. 1990. doi:10.1177/036354659001800408

29. Schmitt H, Lemke JM, Brocai DRC, Parsch D. Degenerative changes in the ankle in former elite high jumpers. *Clin J Sport Med*. 2003. doi:10.1097/00042752-200301000-00002

30. Niek Van Dijk C, Lim LSL, Poortman A, Strübbe EH, Marti RK. Degenerative Joint Disease in Female Ballet Dancers. *Am J Sports Med*. 1995. doi:10.1177/036354659502300307

For peer review only

For peer review only - http://bmjopen.bmj.com/site/about/guidelines.xhtml

31. Murray Leslie CF, Lintott DJ, Wright V. The knees and ankles in sport and veteran military parachutists. *Ann Rheum Dis*. 1977. doi:10.1136/ard.36.4.327

32. Menz HB, Munteanu SE, Landorf KB, Zammit G V., Cicuttini FM. Radiographic classification of osteoarthritis in commonly affected joints of the foot. *Osteoarthr Cartil*. 2007. doi:10.1016/j.joca.2007.05.007

33. Wikstrom EA, Hubbard-Turner T, McKeon PO. Understanding and Treating Lateral Ankle Sprains and their Consequences: A Constraints-Based Approach. *Sport Med*. 2013. doi:10.1007/s40279-013-0043-z

34. Filbay S, Pandya T, Thomas B, McKay C, Adams J, Arden N. Quality of Life and Life Satisfaction in Former Athletes: A Systematic Review and Meta-Analysis. *Sport Med*. 2019;(0123456789). doi:10.1007/s40279-019-01163-0

35. Arnold R, Fletcher D. A Research Synthesis and Taxonomic Classification of the Organizational Stressors Encountered by Sport Performers. *J Sport Exerc Psychol*. 2016. doi:10.1123/jsep.34.3.397

36. Wylleman P, Reints A. A lifespan perspective on the career of talented and elite athletes: Perspectives on high-intensity sports. *Scand J Med Sci Sport*. 2010. doi:10.1111/j.1600-0838.2010.01194.x

37. Nouni-Garcia R, Carratala-Munuera C, Orozco-Beltran D, Lopez-Pineda A, Asensio-Garcia MR, Gil-Guillen VF. Clinical benefit of the FIFA 11 programme for the prevention of hamstring and lateral ankle ligament injuries among amateur soccer players. *Inj Prev*. 2018. doi:10.1136/injuryprev-2016-042267

38. Attwood MJ, Roberts SP, Trewartha G, England ME, Stokes KA. Efficacy of a movement control injury prevention programme in adult men's community rugby union: a cluster randomised controlled trial. *Br J Sports Med*. 2018. doi:10.1136/bjsports-2017-098005

For peer review only

For peer review only - http://bmjopen.bmj.com/site/about/guidelines.xhtml

39. Kirkendall DT, Garrett WE. Management of the Retired Athlete with Osteoarthritis of the Knee. *Cartilage*. 2012. doi:10.1177/1947603511408287

40. McAlindon TE, Bannuru RR, Sullivan MC, et al. OARSI guidelines for the non-surgical management of knee osteoarthritis. *Osteoarthr Cartil*. 2014. doi:10.1016/j.joca.2014.01.003

41. Paterson KL, Gates L. Clinical Assessment and Management of Foot and Ankle Osteoarthritis: A Review of Current Evidence and Focus on Pharmacological Treatment. *Drugs and Aging*. 2019. doi:10.1007/s40266-019-00639-y

42. Gouttebarge V, Goedhart E, Kerkhoffs G. Empowering the health of retired professional footballers: The systematic development of an after Career Consultation and its feasibility. *BMJ Open Sport Exerc Med*. 2018. doi:10.1136/bmjsem-2018-000466

For peer review only

For peer review only - http://bmjopen.bmj.com/site/about/guidelines.xhtml

STROBE Statement—Checklist of items that should be included in reports of ***cross-sectional studies***

	Item No	Recommendation
**Title and abstract**	1	(*a*) Indicate the study's design with a commonly used term in the title or the abstract – √
		(*b*) Provide in the abstract an informative and balanced summary of what was done and what was found – √
**Introduction**		
Background/rationale	2	Explain the scientific background and rationale for the investigation being reported – √: p. 4 line 64 – 96
Objectives	3	State specific objectives, including any prespecified hypotheses – √: p. 4 line 98 – 104
**Methods**		
Study design	4	Present key elements of study design early in the paper – √: p. 6 line 108 – 114
Setting	5	Describe the setting, locations, and relevant dates, including periods of recruitment, exposure, follow-up, and data collection – √: p. 6 line 116 – 133
Participants	6	(*a*) Give the eligibility criteria, and the sources and methods of selection of participants – √: p. 6 line 116 – 133
Variables	7	Clearly define all outcomes, exposures, predictors, potential confounders, and effect modifiers. Give diagnostic criteria, if applicable – √: p. 6 line 135 – 159
Data sources/ measurement	8*	For each variable of interest, give sources of data and details of methods of assessment (measurement). Describe comparability of assessment methods if there is more than one group – √: p. 6 line 161 – 172
Bias	9	Describe any efforts to address potential sources of bias – √: p. 6 line 117
Study size	10	Explain how the study size was arrived at– √: p. 6 – 7 line 130 – 133
Quantitative variables	11	Explain how quantitative variables were handled in the analyses. If applicable, describe which groupings were chosen and why – √: p. 8-9 line 175 – 188
Statistical methods	12	(*a*) Describe all statistical methods, including those used to control for confounding
		(*b*) Describe any methods used to examine subgroups and interactions
		(*c*) Explain how missing data were addressed
		(*d*) If applicable, describe analytical methods taking account of sampling strategy
		(*e*) Describe any sensitivity analyses **– √: p. 8-9 line 175 – 188**
**Results**		
Participants	13*	(a) Report numbers of individuals at each stage of study—eg numbers potentially eligible, examined for eligibility, confirmed eligible, included in the study, completing follow-up, and analysed **– √: p. 10 line 192 – 196**
		(b) Give reasons for non-participation at each stage **– √: p. 10 line 192 – 196**
		(c) Consider use of a flow diagram
Descriptive data	14*	(a) Give characteristics of study participants (eg demographic, clinical, social) and information on exposures and potential confounders **– √: p 11 table 1**
		(b) Indicate number of participants with missing data for each variable of interest
Outcome data	15*	Report numbers of outcome events or summary measures **– √: p 11 – 14, table 2 -4**
Main results	16	(*a*) Give unadjusted estimates and, if applicable, confounder-adjusted estimates and their precision (eg, 95% confidence interval). Make clear which confounders were adjusted for and why they were included **– √: p 11 – 14, table 2 -4**
		(*b*) Report category boundaries when continuous variables were categorized

For peer review only

		(*c*) If relevant, consider translating estimates of relative risk into absolute risk for a meaningful time period
Other analyses	17	Report other analyses done—eg analyses of subgroups and interactions, and sensitivity analyses **– √: p 15, line 227 – 237 + p 16 table 5**
**Discussion**		
Key results	18	Summarise key results with reference to study objectives **– √: p 17, line 242 - 245**
Limitations	19	Discuss limitations of the study, taking into account sources of potential bias or imprecision. Discuss both direction and magnitude of any potential bias **– √: p 3, line 53 - 62**
Interpretation	20	Give a cautious overall interpretation of results considering objectives, limitations, multiplicity of analyses, results from similar studies, and other relevant evidence **– √: p 19 - 20, line 293 - 317**
Generalisability	21	Discuss the generalisability (external validity) of the study results **p 19 - 20, line 293 - 305**
**Other information**		
Funding	22	Give the source of funding and the role of the funders for the present study and, if applicable, for the original study on which the present article is based **p 21, line 339 - 340**

*Give information separately for exposed and unexposed groups.

Note: An Explanation and Elaboration article discusses each checklist item and gives methodological background and published examples of transparent reporting. The STROBE checklist is best used in conjunction with this article (freely available on the Web sites of PLoS Medicine at http://www.plosmedicine.org/, Annals of Internal Medicine at http://www.annals.org/, and Epidemiology at http://www.epidem.com/). Information on the STROBE Initiative is available at www.strobe-statement.org.

For peer review only
