# Supplementary material for: Ankle osteoarthritis and its association with severe ankle injuries, ankle surgeries and health-related quality of life in recently retired professional male football and rugby players: a cross-sectional observational study
Source: BMJ Open. 2020 Jun 21;10(6):e036775. doi: 10.1136/bmjopen-2020-036775 (PMC7310999; doi:10.1136/bmjopen-2020-036775)
Supplement: Reviewer comments [file bmjopen-2020-036775.reviewer_comments.pdf]

## PEER REVIEW HISTORY

BMJ Open publishes all reviews undertaken for accepted manuscripts. Reviewers are asked to complete a checklist review form (**http://bmjopen.bmj.com/site/about/resources/checklist.pdf**) and are provided with free text boxes to elaborate on their assessment. These free text comments are reproduced below.

## ARTICLE DETAILS

| | |
|---|---|
| **TITLE (PROVISIONAL)** | Ankle osteoarthritis and its association with severe ankle injuries, ankle surgeries and health-related quality of life in recently retired professional male football and rugby players: A Cross-sectional observational study |
| **AUTHORS** | Paget, Liam; Aoki, Haruhito; Kemp, Simon; Lambert, Mike; Readhead, Clint; Stokes, Keith; Viljoen, Wayne; Reurink, G; Tol, J; Kerkhoffs, Gino; Gouttebarge, Vincent |

## VERSION 1 – REVIEW

| | |
|---|---|
| **REVIEWER** | Kade Paterson<br>The University of Melbourne, Australia |
| **REVIEW RETURNED** | 18-Feb-2020 |

| | |
|---|---|
| **GENERAL COMMENTS** | The authors have done a good job responding to my previous concerns - well done. |

## VERSION 1 – AUTHOR RESPONSE

Reviewer 1 comment: The authors have done a good job responding to my previous concerns - well done.

Response to reviewer 1: We thank the reviewer for their kind words
